# Returning to Performance After ACL Injury in Competitive Alpine Skiing: A Scoping Review and Evidence‐ and Expert‐Informed Practice Recommendations

**DOI:** 10.1111/sms.70246

**Published:** 2026-03-08

**Authors:** Jörg Spörri, Philippe O. Müller, Christian Fink, Marine Alhammoud, Johannes Scherr, Alli Gokeler, Jan Seiler, Jörg Roten, Marlies Raich, Matthew J. Jordan, Hans‐Christer Holmberg

**Affiliations:** ^1^ Sports Medical Research Group, Department of Orthopaedics, Balgrist University Hospital University of Zurich Zurich Switzerland; ^2^ University Centre for Prevention and Sports Medicine, Department of Orthopaedics, Balgrist University Hospital University of Zurich Zurich Switzerland; ^3^ Gelenkpunkt‐Sports and Joint Surgery FIFA Medical Centre of Excellence Innsbruck Austria; ^4^ Research Unit for Orthopaedic Sports Medicine and Injury Prevention Private University for Health Sciences Medical Informatics and Technology Innsbruck Austria; ^5^ Inter‐University Laboratory of Human Movement Sciences University Claude Bernard Lyon 1 Lyon France; ^6^ Department of Exercise & Health, Exercise Science and Neuroscience Paderborn University Paderborn Germany; ^7^ Faculty of Health Amsterdam University of Applied Sciences Amsterdam the Netherlands; ^8^ Department of Public and Occupational Health, Amsterdam Movement Sciences Amsterdam Collaboration on Health and Safety in Sports Amsterdam the Netherlands; ^9^ Department for Elite Sport Swiss Federal Institute of Sport Magglingen (SFISM) Magglingen Switzerland; ^10^ Swiss‐Ski Worblaufen Switzerland; ^11^ Ski Austria Innsbruck Austria; ^12^ Faculty of Kinesiology, Integrative Neuromuscular Sport Performance Lab University of Calgary Calgary Alberta Canada; ^13^ Faculty of Kinesiology, Sport Medicine Centre University of Calgary Calgary Alberta Canada; ^14^ Division of Machine Elements Luleå University of Technology Luleå Sweden; ^15^ Department of Physiology and Pharmacology, Biomedicum C5 Karolinska Institutet Stockholm Sweden

**Keywords:** athletes, knee injuries, rehabilitation, return to sport, snow sports

## Abstract

Anterior cruciate ligament (ACL) injuries are common in competitive alpine skiing and pose significant challenges for return‐to‐performance (RTP) pathways. The aim of this scoping review was to map the current state of research, identify key factors for recovery, highlight knowledge gaps and synthesize evidence‐ and expert‐informed practice recommendations for RTP after ACL injury. A search was conducted in four electronic databases using a set of pre‐defined search terms. Studies that examined relevant aspects of RTP in competitive alpine skiers (not competitive para alpine skiers or recreational alpine skiers) who had suffered an ACL injury were included. Two assessors independently screened titles, abstracts and full‐text sources to determine eligibility. Of the 622 double‐removed hits from the literature search and the two studies added manually, 35 studies were included in this scoping review based on the eligibility criteria. The entities extracted included study characteristics such as general descriptors, injury patterns, surgical details, rehabilitation/training activities, testing/monitoring metrics, and key outcomes. The extracted data were then narratively/descriptively synthesized across several key domains. Most studies identified focus on epidemiology, secondary risks and early rehabilitation, with limited research on later recovery phases, such as returning to competition and returning to performance. There is also little evidence regarding the psychological and social factors that influence RTP. While further research is needed to fill these knowledge gaps, we propose a sport‐specific, criteria‐based and multi‐domain RTP framework with stepwise progression on snow. Finally, we provide insights into practical implementation and present a comprehensive and jointly created RTP protocol for competitive alpine skiers to serve as a guideline for evidence‐ and expert‐informed practice.

## 1 Introduction

Among Olympic Winter sports, competitive alpine skiing is associated with a particularly high risk of severe knee injury [1], with anterior cruciate ligament (ACL) injuries reported as the most frequent diagnosis [2, 3, 4, 5]. The prevalence of competitive alpine skiers suffering ACL injury per season is as high as 5%–15% [2, 3, 4, 5]. Compared with male skiers, female competitive alpine skiers are more likely to suffer ACL injuries during adolescence (approximately 3.5 times more likely) [6, 7]. At the elite level (i.e., skiers competing at the international level (tier 4) or world class level (tier 5), as defined by McKay et al. [8]), however, the evidence from studies investigating potential sex differences in ACL injury risk is contradictory [2, 4, 9, 10]. While a more recent study reported a higher rate of ACL injuries in female elite skiers [4], previous studies reported no such difference [2, 9, 10].

In competitive alpine skiing, ACL injuries are typically caused by injury mechanisms involving aggressive quadriceps loading that induces anterior tibial translation relative to the femur during jump landing [11, 12, 13], or a combination of dynamic knee valgus with rapid and high‐force quadriceps contraction, which induces additional tibial internal rotation when the ski abruptly catches its inner or outer edge while turning [11, 14, 15]. Compared with the ACL injury mechanisms in field and court sports, the forces and kinetic energy involved in ACL injury mechanisms in competitive alpine skiing are particularly high [15, 16]. Furthermore, in a recent comprehensive systematic review categorizing ACL injury situations across sports, those in alpine skiing were identified as being predominantly “gear‐induced,” meaning that the equipment influences the transmission of adverse forces from the ski–snow interaction to the knee [17]. Such equipment involvement makes the acting forces and their transmission to the knee less predictable and controllable for athletes [17].

Owing to their severity and complexity, ACL injuries must be considered career‐threatening events for competitive alpine skiers, with both short‐term effects, such as surgery and long periods of absence [18], and long‐term effects, such as an increased risk of ACL reinjury and/or osteoarthritis [19, 20]. However, despite the urgent need for improved return‐to‐sport (RTS)/return‐to‐performance (RTP) practices, the pathway back to elite performance remains inadequately defined. Resuming competitive alpine skiing after ACL injury entails high‐risk‐taking demands, such as high‐speed skiing and high‐amplitude jumps. These activities pose substantial biomechanical/physical, psychological, and social challenges that require a holistic approach before athletes return to sport [21]. Accordingly, recent work advocates for a biopsychosocial, athlete‐centered RTS framework that explicitly incorporates psychological readiness for risk‐taking in snow sports [21, 22]. However, while surgical and rehabilitation advances have improved clinical outcomes following ACL injuries in recent decades [23, 24, 25, 26], particularly with respect to the full RTP journey (i.e., the phases beyond the RTS decision), a significant knowledge gap remains in the transition from athlete's clinical surgery and rehabilitation‐supported RTS to full sport‐specific training‐supported RTP in competitive alpine skiing.

For example, one study reported that while 90% of skiers returned to their previous competition level, only 16%–33% improved their world ranking by 3 years after ACL reconstruction, whereas 60% of uninjured controls did [27]. This aligns with broader observations of persistent fitness and knee function deficits in competitive skier cohorts even after RTS clearance [28]. Moreover, ACL injury is associated with neuroplastic changes that influence perceptual‐motor‐cognitive function—a factor of immense importance in cognitively demanding sports such as competitive alpine skiing [29]. Finally, concrete recommendations for returning to snow and the corresponding progress toward a full RTP are limited to a few examples [30, 31, 32]. Thus, the sport‐specific requirements of successful RTP pathways are rarely documented in the literature, despite their major relevance for athletes recovering from ACL injuries in competitive alpine skiing.

Therefore, this scoping review addresses the urgent need for sport‐specific and evidence‐ and expert‐informed practice recommendations. The specific aims were (1) to systematically map the existing scientific literature on the RTS and RTP continuum following ACL injury in competitive alpine skiers; (2) to identify and synthesize the biomechanical/physical, psychological, and social determinants of full performance recovery, both on and off the snow, along with the corresponding assessment metrics; (3) to highlight critical knowledge gaps within the current evidence, as well as unmet needs regarding future research and practical implementation; and (4) to develop evidence‐ and expert‐informed practice recommendations, as well as to jointly create a sport‐specific protocol to guide RTP following ACL injury in competitive alpine skiing.

## 2 Materials and Methods

### 2.1 Study Protocol, Reporting and Registration

This study was conducted as a scoping review. The Preferred Reporting Items for Systematic Reviews and Meta‐Analysis Extension for Scoping Reviews (PRISMA‐ScR) [33] and the Joanna Briggs Institute (JBI) Guidelines on Scoping Reviews [34] were used. The underlying study protocol was preregistered in the Open Science Framework (OSF) database (https://osf.io/dvr5t).

### 2.2 Data Sources, Search Strategy and Eligibility Criteria

A comprehensive literature search was conducted in MEDLINE/PubMed, EMBASE, Web of Science, and Scopus. The search was performed on 15 September 2025. The exemplary search string used in EMBASE is presented in Table 1. The eligibility criteria using the Population, Intervention, Comparison, Outcome, Context (PICOC) methodology are illustrated in Table 2. A search validation procedure was employed to define this final search string. The criterion was that at least one database had to have identified 25 preselected key articles in the field, as defined by the authors on the basis of their personal literature overview. Studies were considered if they were peer‐reviewed original research published in English or German within the last 30 years. This timeframe coincides with the introduction of carving skis in competitive alpine skiing and the period during which ACL injury patterns have remained similar to those observed today. As outlined in the PICOC description (Table 2), the considered studies had to be “original studies” that reported either quantitative or qualitative data and could be either observational or interventional in design. “Current practice descriptions” (defined as what is currently being done in practice) can be considered qualitative evidence, which is why they were included. Reviews and gray literature were excluded to ensure that the synthesis relied solely on peer‐reviewed primary data. As no meta‐analysis was conducted, there was no need to contact the authors. Owing to the low frequency of publications on the topic, no repetition of the search is planned. In addition to this main search strategy, the reference lists of all included articles were manually screened, and targeted searches were performed on key authors known for their work in this field (Jordan MJ, Spörri J, Smith C) to identify any further relevant publications.

**TABLE 1:** Search strategy used in EMBASE.

No.	Query
#1	(‘skiing’/exp. OR ‘skier’/exp) AND (professional*:ti, ab, kw OR elite:ti, ab, kw OR competitive:ti, ab, kw OR athlete*:ti, ab, kw OR racer*:ti, ab, kw OR racing:ti, ab, kw OR ‘high performance’:ti, ab, kw OR ‘high speed’:ti, ab, kw) OR (‘elite athlete’/exp. AND (ski:ti, ab, kw OR skiing:ti, ab, kw OR skier*:ti, ab, kw OR ‘snow sport*’:ti, ab, kw OR snowsports:ti, ab, kw)) OR ((racing:ti, ab, kw OR racer*:ti, ab, kw OR elite:ti, ab, kw OR competitive:ti, ab, kw OR professional*:ti, ab, kw OR athlete*:ti, ab, kw OR ‘high performance’:ti, ab, kw OR ‘high speed’:ti, ab, kw) AND (ski:ti, ab, kw OR skier*:ti, ab, kw OR skiing:ti, ab, kw)) OR ‘snow sport*’:ti, ab, kw OR snowsports:ti, ab, kw
#2	‘anterior cruciate ligament injury’/exp. OR ‘anterior cruciate ligament reconstruction’/exp. OR ‘anterior cruciate ligament’/exp. OR ‘knee ligament injury’/de OR ‘return to sport’/exp. OR (((injur* OR reconstruct*) NEAR/6 (knee OR acl OR ‘anterior cruciate ligament’ OR ‘anterolateral ligament’)):ti, ab, kw) OR leap:ti, ab, kw OR ‘lower extremity assessment protocol’:ti, ab, kw OR rehabilitation:ti, ab, kw OR ‘limb symmetry index’:ti, ab, kw OR ‘rate of force development’:ti, ab, kw OR stability:ti, ab, kw OR (((‘return to’ OR ‘back to’) NEAR/6 (activit* OR sport* OR play OR snow OR ski OR skiing OR competition* OR performance)):ti, ab, kw)
#3	#1 AND #2
#4	#3 AND [1995–2025]/py
#5	#4 AND ([english]/lim OR [german]/lim)
#6	#5 NOT ([conference abstract]/lim OR [editorial]/lim)

**TABLE 2:** Eligibility criteria.

Dimension	Eligibility criteria
Population	Competitive (not recreational) alpine skiers of any age or sex/gender who had sustained an ACL injury, either in isolation or as part of a multi‐ligament knee injury. Surgical ACL reconstruction was not necessary for inclusion
Intervention	Original studies that reported either quantitative data or qualitative data (including current practice descriptions); both observational or interventional in design
Comparison	Controlled and noncontrolled studies
Outcome	Studies that reported on outcomes such as descriptive data, experiences, or protocols related to RTS or RTP, or presented physical, psychological and social determinants of performance recovery, both on and off the snow, along with the corresponding assessment metrics
Context	Studies focusing on rehabilitation or training following ACL or multi‐ligament knee injuries

Abbreviations: ACL, anterior cruciate ligament; RTP, return‐to‐performance; RTS, return‐to‐sport.

### 2.3 Study Selection Process

All identified studies were screened for relevance via a two‐step approach. In the first step, following the removal of duplicates, two assessors (J. Sp., P. O. M.) independently screened all titles and abstracts for relevance using Rayyan [35]. Rayyan is a web‐based tool that helps researchers optimize the process of literature reviews by effectively handling, evaluating (title/abstract/full‐text), and extracting information from research studies. In a second step, the full texts of potentially eligible articles were then retrieved and assessed independently by the same two assessors for final inclusion. There were no specific instructions to the assessors and no blinding of any bibliographic fields during the study selection process. Blinding was considered inefficient, given the small number of research groups and studies focusing on the subject matter of this scoping review. Any discrepancies between the two assessors during the screening process were resolved through discussion and consensus. The selected studies, in which the two assessors (J. Sp. and P. O. M.) were part of the authorship, were reconfirmed for inclusion by a third independent assessor (M. J. J.). All studies selected through the screening procedure were included in this explorative scoping review. The results of the search and selection process are illustrated in a PRISMA flow diagram.

### 2.4 Study Analysis Process

The analysis of the identified studies included (1) data extraction using a pre‐defined charting form and (2) a narrative/descriptive synthesis of the data, providing a qualitative summary of the studies' major findings and remaining knowledge gaps.

Data extraction was performed by one accessor (J. Sp.), whereas a second assessor (P. O. M.) extracted the data from the first five studies to verify the reliability and completeness of the extraction. As there were no relevant disagreements, J. Sp. subsequently extracted the data from the remaining studies. There was no extractor masking. The assessor(s) conducting the data extraction did not receive any specific instructions, except for a pre‐defined custom‐developed charting form in Excel (Version 2019, Microsoft, US). The charting form was developed by the article's first and second authors (J. Sp., P. O. M.) and was, after data extraction from five articles, jointly reevaluated, whereas no adaptations were necessary. The entities extracted included study characteristics such as general descriptors (study title, authors, year of publication, study design, sample size, and patient characteristics), injury patterns (type, severity, concomitant injuries, previous injuries to the same structure), surgical details, rehabilitation/training activities, testing/monitoring metrics, and key outcomes such as athlete proportions with timelines related to RTS/RTP, patient‐reported outcome measures (PROMs), off‐ and on‐snow performance outcomes, and implementation barriers and facilitators. If available, rates of reinjury, complications, or revision surgery were also reported.

The narrative/descriptive data synthesis was then performed again by J. Sp., and as part of the final manuscript approval process, all coauthors provided their feedback on the qualitative summary of the major study outcomes and their interpretation. As there was no disagreement with the narrative/descriptive data synthesis and interpretation by any of the coauthors, no further resolving actions were needed. The charted data were narratively/descriptively summarized across several key domains, which were developed inductively based on the main groups of themes described in the identified studies and expanded deductively based on the pre‐defined study objectives. These domains included clinical outcomes and secondary injury risk, performance outcomes and RTS/RTP rates, as well as the determinants and metrics of successful RTS/RTP, multimodal RTS/RTP protocols and implementation aspects. In addition, any missing data were reported, and the corresponding findings were subsequently used to identify knowledge gaps and to develop an evidence‐ and expert‐informed RTP protocol for competitive alpine skiers following ACL injury. All studies that were identified and passed through the screening procedure served as the basis for drawing conclusions. The corresponding conclusions were critically reflected in terms of their theoretical reasonability and in view of the existing body of general knowledge from different sports. There was synthesis blinding. As this is a scoping review describing and mapping the current body of literature in terms of characteristics and factors detailed by the review's objective, the methodological quality assessment of the selected articles was omitted [34].

### 2.5 Development of Expert Recommendations

In the final phase, the authors of this scoping review synthesized the major outcomes of the included studies to formulate key practice recommendations. By integrating these recommendations within the structural RTP framework from the recent International Ski and Snowboard Federation (FIS) consensus statement [36], the authors then developed an evidence‐ and expert‐informed RTP protocol for competitive alpine skiers following ACL injury. This protocol combines the evidence gathered in this scoping review with the multidisciplinary end‐user expertise of the author group: orthopedic surgery (C.F.), sports medicine (M. A. and J. Sc.) physiotherapy (A. G.), strength and conditioning (J. Se. and M. J. J.), sport science (M. J. J., J. Sp. and H. H.), sports psychology (P. O. M.), on‐snow coaching (J. R. and J. Sp.) and former athletes (M. R. and J. Sp.). Finally, the authors compiled practical strategies addressing neuromuscular, psychosocial, and perceptual‐cognitive readiness, alongside specific on‐snow progressions and clinical/practical implementation insights.

## 3 Results

### 3.1 Selected Studies

**FIGURE 1:**
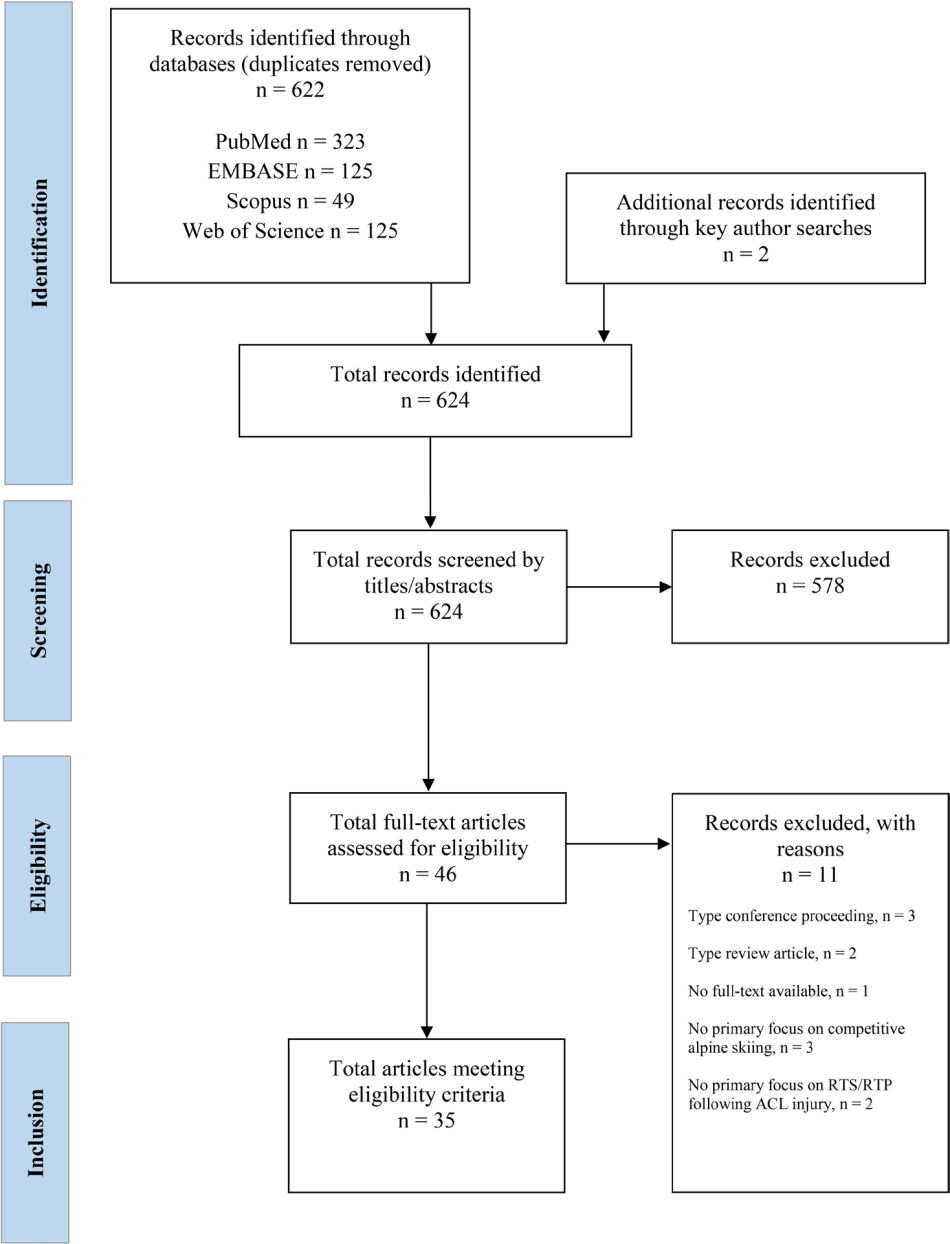
Preferred reporting items for systematic reviews and meta‐analyses extension for scoping reviews (PRISMA‐ScR) flow diagram (*n* = the number of studies). ACL, Anterior cruciate ligament; RTP, Return‐to‐performance; RTS, Return‐to‐sport.

Figure 1 shows a flow diagram that describes the study selection process in more detail. Among the 622 duplicate‐cleaned hits from the literature search and the 2 manually added studies, 578 were excluded on the basis of the aforementioned eligibility criteria during title and abstract screening, and 11 were excluded during full‐text assessment. This resulted in 35 studies being included and considered for subsequent data extraction and synthesis.

### 3.2 Extracted Data

The full details of the extracted data of the 35 studies selected for inclusion are presented in File S1.

Among the selected studies, 31 (88.6%) were “original,” including 26 (74.3%) quantitative studies and five (14.3%) qualitative studies. Four (11.4%) were “non‐original” clinical best practice descriptions. The quantitative studies were primarily case studies (*n* = 7 studies; 20.0%), retrospective studies (*n* = 10 studies; 28.6%), or cross‐sectional studies (*n* = 6 studies; 17.1%). Only three studies (*n* = 3 studies; 8.6%) used prospective data collection protocols. The qualitative studies were all semi‐structured stakeholder interviews. The quantitative studies included data from 4529 alpine skiers and the qualitative studies examined the perceptions of 62 expert stakeholders. Across all the studies, the participants were primarily competitive alpine skiers of various levels and ages.

In terms of injury patterns, six studies (17.1%) focused on competitive skiers with severe injuries, including ACL injuries; 11 studies (31.4%) examined skiers with ACL injuries and reconstruction; and nine studies (25.7%) compared skiers with ACL injuries and reconstruction with healthy controls. Five studies (14.3%) reported on individual complex injury cases, including ACL injuries accompanied by severe concomitant injuries. Four studies (11.4%) examined skiers with secondary ACL reinjury. Nine of the selected studies (25.7%) explicitly mentioned that ACL injuries were accompanied by multi‐ligament injuries, meniscal involvement, cartilage damage, or tendon injury.

Sixteen (45.7%) of the studies reported the surgical details of the ACL reconstructions performed. Only six (17.1%) of the studies reported details of the rehabilitation/training activities performed, and 14 (40%) of the studies reported details of the testing/monitoring metrics assessed. While most studies (82.9%) focused on the physical aspects of RTP, only six studies (17.1%) addressed psychological or social aspects. The main outcomes reported included ACL injury and reconstruction rates (*n* = 6 studies; 17.1%), athlete proportions with timelines related to RTS/RTP (*n* = 2 studies; 5.7%), rates of reinjury or revision surgery (*n* = 4 studies; 11.4%), patient‐reported outcome measure (PROM) scores (*n* = 1 study; 2.9%), off‐ and on‐snow performance outcomes (*n* = 8 studies; 22.9%), and best practice recommendations including implementation barriers and facilitators (*n* = 14 studies; 40%). Seven studies (20.0%) reported results that could theoretically be assigned to more than one of the above categories; however, the authors assigned them to only one main category, which could be subject to a certain degree of subjectivity.

### 3.3 Narrative/Descriptive Synthesis of the Key Outcomes of the Studies Identified

The subsequent paragraphs summarize and interlink the key outcomes of the 35 selected studies using a narrative/descriptive synthesis approach. The subheadings thereby represent the major themes covered by the corresponding studies.

#### 3.3.1 Clinical Outcomes and Secondary ACL Injury Risk

According to the studies identified in this scoping review, ACL injuries in competitive skiers are common and complex, as are secondary ACL injuries [9, 19, 37, 38]. The mean age at the time of primary ACL injury has been reported to be approximately 21.6 ± 3.5 years [27]. A majority (up to 82%) of ACL injuries are accompanied by concomitant injuries involving multiple‐ligament injuries, such as those to the ACL combined with the medial collateral ligament (MCL), as well as lateral compartment chondral lesions and complex meniscal injuries, both of which often further worsen over time [37]. In other cohorts, multi‐ligament, meniscal, and chondral pathologies were likewise frequent, underscoring the clinical complexity of causation and recovery [27, 38]. Accordingly, patient‐reported outcomes after ACL reconstruction were reported to be slightly poorer than those reported in other high ACL risk sports, such as basketball, football or soccer [39]. Nevertheless, even complex ACL injuries, such as ACL injuries with simultaneous locked bucket–handle tears of both the medial and lateral meniscus, may have good prospects, provided that appropriate surgical treatment is applied [40].

Primary ACL injuries have been shown to substantially increase the risk of a second ACL injury [9]. Following primary ACL injury, athletes face a substantial risk of secondary ACL injury, with reported rates in elite competitive alpine skiing cohorts ranging from 19% to 47% [9, 19, 37]. Sex and time to return‐to‐competition are not significantly associated with the risk of secondary ACL injury [19]. Contralateral tears are a major contributor to this burden and, in some cohorts, occur more frequently than ipsilateral re‐ruptures do (e.g., 30.0% contralateral vs. 16.7% ipsilateral) [19, 41]. Revision ACL procedures are required in approximately 22%–28% of cases [7, 37]. Surgical techniques appear to influence the risk of graft rupture; for example, adding a lateral extra‐articular procedure (LEAP) to ACL reconstruction was shown to significantly reduce graft rupture rates in elite French skiers from 34.0% to 6.5% [41]. Moreover, combined ACL repair with anterolateral ligament (ALL) reconstruction appears to offer important advantages with respect to graft rupture rates, return to sport, knee stability, and reoperation rates after injury, particularly if a quick return to competition is the main intention [42, 43].

Age and graft choice may also be critical risk factors for ACL graft rupture, whereas there is contradicting evidence in the literature for competitive alpine skiers [41, 44]. At least large registry data indicate markedly higher revision rates in younger athletes, with skiers aged ≤ 18 years facing a fivefold greater risk than those aged ≥ 35 years [44]. The same data revealed that hamstring tendon autografts are associated with a 1.8‐fold greater hazard for revision than bone‐patellar tendon‐bone grafts are, a risk that increases 2.8‐fold in the adolescent (≤ 18 years) population [44]. A functional explanation for this increased risk could be the observation that athletes who underwent semitendinosus (ST) tendon autografting for anterior cruciate ligament reconstruction showed persistent deficits in maximum knee flexion torque 1–3 years after surgery, despite returning to competitive sports without restrictions [45]. Considering that maximal, eccentric, and rapid activation of the hamstrings play crucial roles in controlling the load on the ACL during typical injury mechanisms in competitive alpine skiing, this may represent critical functional impairment, increasing the risk of reinjury [46]. Like the risk of a second ACL injury, sex has not yet been found to be a risk factor for ACL graft rupture in competitive alpine skiers [41].

#### 3.3.2 Performance Outcomes and RTS/RTP Rates

The evidence identified in this study suggests that competitive alpine skiers can return to sports and achieve preinjury performance or even greater performance following ACL (re‐)injuries [27, 28, 47]. Crucially, returning to sport or a specific competition level does not necessarily equate to returning to performance [27]. According to a recent study of 30 high‐level competitive alpine skiers, 90% of the ACL‐injured skiers were able to return to their preinjury competition level [27]. However, compared with their preinjury baseline values, only 53% improved in FIS points, and 43% improved in FIS ranking in one of the speed or technical disciplines by 3 years after surgery [27]. Moreover, when propensity score matching was used to pair skiers with ACL injuries with matched healthy controls, 63% of healthy control skiers improved their FIS ranking at the 3‐year mark, whereas 16% of speed skiers and 39% of technical skiers who were post‐ACL reconstruction improved their FIS ranking [27]. In this context, the age at which ACL injury occurs seems to play a crucial role; skiers whose performance improved after ACL injury are typically younger (men, 22.2 ± 3.0 years; women, 18.7 ± 2.2 years) at the time of injury than those whose performance deteriorated after ACL rupture (men, 25.3 ± 4.2 years; women, 22.4 ± 4.0 years) [47]. In contrast, athletes' career length was not found to be reduced because of ACL injuries [9]. For example, despite their higher ACL injury rate, skiers ranking in the Top 30 of the world‐ranking lists had longer careers than the other skiers did [9].

The typical return‐to‐competition timeline is approximately 12 months (mean 364 ± 143 days) post‐ACL reconstruction [28]. However, timelines may vary significantly across individuals and their specific injuries; some complex injuries may result in a safe and timely return to preinjury level performance within 14 months [32], whereas others may require multiple surgeries that dramatically prolong the return to competition, with one case report detailing a three‐year journey back to elite‐level performance [48]. Similarly, an exceptional return to the Olympics was documented at just 5 months following combined ACL repair and anterolateral ligament reconstruction [42], whereas in another complex case involving multiple ligaments, the skier only returned to restricted training sessions on snow after 7 months [32]. Thus, generally valid forecasts for RTS or RTP times cannot be reliably provided; therefore, it must be clearly communicated to injured athletes that these timelines are not guaranteed, which is crucial for fostering realistic expectations.

#### 3.3.3 Determinants and Metrics of Successful RTS/RTP

To ensure safe and successful RTS and RTP in competitive alpine skiing, a variety of biomechanical/physical, psychological and social (bio‐psycho‐social) determinants need to be considered [46]. The corresponding processes require reasonable goals and clear structures, including milestones and metrics for evaluation purposes [22]. In this context, a recent study revealed the importance of sport performance practitioners and clinicians using distinct criteria instead of only considering the time to progress rehabilitation after an ACL injury [49]. This may include objective monitoring and testing to determine readiness for the next RTS/RTP phase, covering physical, psychological, social, and perceptual–motor–cognitive functions.

##### 3.3.3.1 Physical Determinants and Metrics

Despite their RTS or RTP, elite skiers commonly show persistent neuromuscular deficits that extend throughout ACL rehabilitation and beyond [28, 45, 50, 51, 52]. These deficits may include muscle morphology impairments [45], general strength and power deficits [28, 53], asymmetries in quadriceps and hamstring strength [51, 53], a reduced rate of force development (RFD) [51], and impaired/asymmetric explosive characteristics or thigh muscle coactivity during jump tasks [54, 55]. Furthermore, such deficits may predispose competitive alpine skiers to a second ACL injury, as reported in recent observational studies of adolescent competitive alpine skiers [56]. However, most critically, these deficits are long‐lasting; for example, even when assessed 5–6 months post‐ACL reconstruction and beyond, knee extensor and flexor strength, as well as power deficits, may still be evident [28, 53]. Moreover, stretch‐shortening cycle (SSC) capacity (e.g., countermovement jump height and peak power) can remain depressed for up to 5 years after surgery, even as between‐limb asymmetry appears to normalize [52].

These observations clearly support the use of advanced, SSC‐oriented force–plate metrics for the longitudinal monitoring of neuromuscular function in skiers throughout their careers and their recovery from ACL injuries [54, 57]. Accordingly, and in view of the other factors typically involved in ACL injury mechanisms (e.g., aggressive quadriceps loading and/or a combination of dynamic knee valgus and tibial internal rotation) and the events leading to ACL injury situations (e.g., out‐of‐balance situations inwards or backwards), the following metrics should be considered essential RTS/RTP monitoring and/or testing metrics: knee muscle strength capacities (especially maximal and explosive strength (RFD), as well as SSC capacity and eccentric control); limb asymmetries; intermuscular coordination patterns (especially between hamstrings and the quadriceps); and potentially measures of dynamic postural control, core stability and dynamic knee valgus control [46].

In contrast to neuromuscular impairments primarily induced by muscle morphology and muscle function, a case study of a 16‐year‐old female competitive skier reported reflex extension loss due to femoral high‐noon graft placement [58]. Despite the rarity of such cases, surgical failure should also be considered as a differential diagnosis when evaluating patients with an extension deficit after ACL reconstruction, particularly if the impairments persist over a long period of time and do not improve [58].

##### 3.3.3.2 Psychological Determinants and Metrics

The psychological and social components of recovery are critical determinants of successful RTP in competitive alpine skiing [22]. This underscores the need for a holistic, biopsychosocial, and interdisciplinary RTS framework that explicitly integrates an athlete's psychological readiness and social context with their physical recovery [21]. Qualitative studies with elite athletes and stakeholders reinforce this, revealing that fear of reinjury, kinesiophobia, fluctuating confidence, and a perceived lack of support act as significant barriers, whereas strong self‐belief, well‐coordinated expert support, specialized infrastructures and available resources are key facilitators [22, 59, 60]. While general (non‐skiing‐related) evidence suggests that injuries pose significant psychological challenges for severely injured athletes, a key factor for successful rehabilitation is the athlete's ability to maintain a positive attitude and mindset during recovery [46]. For example, injured skiers may see season‐ending injuries as opportunities for personal growth, psychologically based performance enhancement, and further development of physical‐technical skills [61].

Typically, information processing, reactive behavior, and coping attempts are key responses of skiers to severe injuries [61]. In this context, research on competitive alpine skiers suggests that improving resilience against injury‐related stressors, rebuilding confidence, managing emotions such as fear when resuming risk‐taking, and engaging in shared decision making may be important determinants of athletes' successful RTS and RTP journeys [46, 62]. The two available literature sources on practices in competitive alpine skiing suggest the use of cognitive and behavioral coping strategies [46, 62], such as increasing mental toughness, athletes' sense of control, and self‐compassion, which are important factors to work on, as are acceptance, long‐term focus, and viewing rehabilitation as a form of athletic performance enhancement [46]. Moreover, imagery has been suggested to be one of the most effective, evidence‐based tools available for injury rehabilitation [46]. However, the literature on the psychological determinants of RTS/RTP in competitive alpine skiers is notably limited, and clear metrics for monitoring and testing are lacking.

##### 3.3.3.3 Social Determinants and Metrics

Two studies identified in this scoping review revealed that social support can be particularly important in overcoming the major challenges associated with recovery from severe injuries [59, 63]. In this context, competitive alpine skiers need various types of support in their RTS/RTP journeys, including emotional support (e.g., empathy, active listening and encouragement), informational support (e.g., diagnoses, feedback, information about the experiences of others and acknowledgement of effort) and tangible support (e.g., access to an expert network and a well‐organized support team) [63]. In addition to the social support of an interdisciplinary RTS/RTP team, family support is considered particularly important [59]. Another qualitative study with stakeholders from competitive snow sports revealed that providing a safe, athlete‐centered social environment on the basis of trust was considered a key factor in the successful return to RTS/RTP [22]. The competitive snow‐sport setting is, however, characterized by a high degree of geographic decentralization, seasonal climatic limitations, and alternations of training in off‐snow and on‐snow settings (and therefore different organizational and social settings). This makes standardized social support challenging and reinforces the need for holistic and integrative approaches that consider determinants and metrics from a true biopsychosocial perspective [21]. However, despite the recognition of the importance of social determinants of RTS/RTP success among competitive alpine skiers, no specific evaluation approaches or metrics have yet been proposed in the literature.

##### 3.3.3.4 Perceptual‐Motor‐Cognitive Function Determinants and Metrics

Complementing the aforementioned evidence on biopsychosocial determinants and metrics, a distinct but related finding is that ACL injury is associated with neuroplastic changes that affect perceptual–motor–cognitive control [29]. Current paradigms largely neglect this, even though skiers experience an elevated dual‐task cost and must process a high cognitive load in a variable, high‐speed environment [29]. It is therefore argued that RTS testing and on‐snow progress should integrate dual‐task and sensory‐challenge paradigms to better prepare athletes for these on‐snow demands [29]. For example, recommendations include additional perceptual–motor–cognitive tasks into standard RTS/RTP tests and the quantification of metrics related to reaction times, motor performance and movement quality with or without cognitive load or the use of sports equipment to better replicate the sport's perceptual and cognitive demands during testing [29]. One may also consider distinct return‐to‐snow progressions, including perceptual, motor and cognitive elements or even drills that challenge proprioception while on snow; these may help to enhance current RTS/RTP protocols for competitive alpine skiers [29].

#### 3.3.4 Multimodal RTS/RTP Protocols

Despite the limited availability of evidence‐based, multimodal protocols and case studies describing the entire RTS/RTP journey of competitive alpine skiers, there are some key literature resources on the subject [30, 31, 32, 42, 48, 49]. What they have in common is criterion‐based and phase‐specific progression from the time of injury until full RTS or even RTP [30, 31, 32, 42, 48, 49]. While there are more concrete recommendations that can be made for the postsurgery, early rehab, mid rehab, and late rehab phases [30, 31, 32, 42, 48, 49], there are fewer sources that suggest concrete off‐ and on‐snow training drills beyond RTS clearance. Moreover, the clinical and physical aspects of RTS/RTP are clearly overrepresented [30, 31, 32, 42, 48], whereas only one study has attempted to follow a more holistic and multimodal approach from a biopsychosocial perspective and foster interprofessional collaboration [49].

#### 3.3.5 Implementation Aspects

From an implementation perspective, qualitative research findings suggest that transparent communication for better decision‐making, the lack of objective sport‐specific criteria, and the coordination of available resources and expert network structures are practical challenges that influence the RTS/RTP process, which should be tailored to each athlete to achieve a successful outcome [22]. This may require superior interprofessional collaboration, clear roles and responsibilities for all involved, as well as well‐structured and organized RTS/RTP frameworks and communication processes/tools [22]. Accordingly, incorporating an interprofessional RTS/RTP with clear communication between the athlete, surgeon, physiotherapist, strength and conditioning coach, sport psychologist, and technical coach is essential throughout the entire process.

## 4 Discussion

### 4.1 What Do We Know, and Where Do We Go From Here?

This scoping review systematically maps the existing scientific literature on the RTS and RTP continuums following ACL injury in competitive alpine skiers. Most of the studies either described the ACL epidemiology and secondary injury risk along with average clinical and/or performance prospects following RTS/RTP [7, 9, 19, 27, 28, 37, 38, 39, 40, 41, 43, 44, 47, 52], the implementation context [22, 46, 59], or provided evidence‐informed recommendations for the early and mid‐rehabilitation phases [40, 42, 48]. However, despite evolving evidence over the last decade, especially regarding the later rehabilitation and training phases following return to activity (i.e., return‐to‐snow, return‐to‐sport, return‐to‐competition and return‐to‐performance), very little evidence exists [30, 31, 32, 49]. Moreover, most studies have focused on the physical determinants of performance recovery, whereas studies on the psychological and social aspects of RTS and RTP are widely lacking [22, 59, 60, 61, 62, 63]. Both future research efforts and the development of evidence‐based guidelines are warranted.

### 4.2 Evidence‐Informed Recommendations for RTP Practice

The evidence identified in this review strongly advocates a shift away from simplistic, isolated criteria and toward a multi‐domain, criteria‐based RTP framework. Such a framework may be organized into the specific, progressive phases “post‐injury,” “presurgery,” “postsurgery,” “early rehab,” “mid rehab,” “late rehab,” “early team training,” and “regular team training” (as defined by [36]) and, according to the key findings of this scoping review, it is suggested that it integrates assessments across several key areas:

1. Neuromuscular readiness: Neuromuscular training and testing are recommended to go beyond simple inter‐limb symmetry to include measures of knee muscle strength capacity that better represent skiing's unique demands (especially maximal and explosive strength (RFD), SSC capacity and eccentric control) and, where relevant, measures of dynamic postural control, core stability and dynamic knee valgus control [28, 45, 46, 50, 51, 52, 53, 54, 55, 64]. Critically, absolute performance should be benchmarked against preinjury data or sport‐specific norms because acceptable symmetry can mask bilateral deficits in function [46, 49].
2. Psychological readiness and social support: Psychological training and testing should include improving and formally assessing an athlete's confidence, kinesiophobia, fear of reinjury, and perceived support [46, 59, 60, 62]. This should occur within an athlete‐centered, interdisciplinary process that fosters a safe and supportive environment and prioritizes shared decision‐making [22, 46, 59, 63].
3. Perceptual‐motor‐cognitive readiness: Rehabilitation, training and testing on the way to RTP are also recommended to involve perceptual‐motor‐cognitive challenges, such as integrating dual‐task and reactive paradigms to assess and train an athlete's ability to maintain motor control while under cognitive load [29]. This includes incorporating unanticipated stimuli and visual challenges within both functional tests and on‐snow drills [29].
4. On‐snow progression: A gradual on‐snow progression protocol that is coordinated across all stakeholders is essential [30, 31, 32]. Such progression should advance systematically from controlled, restricted drills on groomed terrain, through increasingly complex and variable conditions, to full race‐simulation intensity until full on‐snow performance competence has been restored [30, 31, 32, 49].
5. Longitudinal monitoring/testing: Given that recovery can be prolonged and elite skiers face a substantial secondary injury burden in the first few seasons after return, a plan for continuous monitoring is necessary [49]. This should include tracking workload, neuromuscular function, and on‐snow performance, particularly while known SSC and neuromuscular deficits persist [28, 46, 49, 53, 55]. Moreover, psychological monitoring is crucial, as it provides insight into an individual's emotional state and can help prevent adverse events from occurring [46, 49, 60].

### 4.3 Expert‐Informed Recommendations for RTP Practice

On the basis of the aforementioned evidence‐informed recommendations for RTP practice, Tables 3, 4, 5, 6, 7, 8, 9, 10 present an evidence‐ and expert‐informed RTP protocol tailored to the demands of competitive alpine skiers following ACL injury that was jointly created by the authors, including key measures and exercises for the dimensions of neuromuscular readiness, psychological readiness and social support, perceptual–motor–cognitive readiness, and on‐snow progression across all relevant RTS/RTP phases. Moreover, File S2 contains a collection of practice ideas for work in the RTS/RTP dimensions of neuromuscular readiness, psychological and social readiness, perceptual‐motor‐cognitive readiness, and on‐snow progression. In addition, File S3 provides eight illustrative clinical/practical experience reports from the perspectives of an orthopedic surgeon, a sports physician, a physiotherapist, a strength and conditioning expert, a sports scientist, a sports psychologist, an on‐snow coach, and a former athlete. With these expert‐informed practice guidelines, we hope to guide more systematic RTP pathways in competitive alpine skiers and to stimulate further research efforts in the field.

**TABLE 3:** Evidence‐ and expert‐informed RTP protocol tailored to the demands of competitive alpine skiers following ACL injury—Phase 1.

Phase 1: Post‐injury	
Timeline: From injury until a definitive medical plan is established	
Overall goals	Diagnose & plan: obtain an accurate diagnosis (clinical exam, MRI) and decide on a treatment plan together with the athlete (quick surgery is not always best) Control symptoms: manage acute pain and effusion to protect the knee joint, utilizing crutches and bracing as indicated; RICE (rest, ice, compression, elevation)/heat therapy Establish the RTP team, assign responsibilities and define communication pathways Consult/educate the athlete: provide clear information on the injury, process, and timeline to manage expectations
Neuromuscular readiness	Restore foundational ROM: prioritize regaining full passive knee extension and achieving at least 90° of flexion as swelling permits; restrict general activity and articular knee ROM as appropriate Maintain global strength: continue contralateral limb, core, and upper‐body conditioning
Psychological & social readiness	Address the initial psychological impact: manage emotions and acute stress Foster acceptance; process the event/incident Build or activate a social support network and/or peer support Maintain identity: foster regular, meaningful contact with the team to preserve the athlete's social connection and identity The athlete must be and remain communicative and discuss everything that occupies their mind
Perceptual‐motor‐cognitive readiness	n/a
On‐snow progression	n/a
Monitoring & testing	Clinical baseline assessment: orthopedic tests (e.g., Lachman, Pivot Shift) and confirmation with MRI Effusion/pain/arthrogenic inhibition assessment: stroke test, knee circumference, and VAS Morphological baseline assessment: body composition (DXA, anthropometric testing) Physical baseline assessment: quantify ROM via goniometric measurement Psychological baseline assessment: assess mental health using the SMHAT‐1 or a similar tool; measure baseline psychological readiness using the ACL‐RSI scale

Abbreviations: ACL‐RSI, ACL‐return to sport after injury scale; DXA, dual‐energy X‐ray absorptiometry; MRI, magnetic resonance imaging; ROM, range of motion; RTP, return‐to‐performance; SMHAT‐1, sport mental health assessment tool, version 1; VAS, visual analogue scale.

**TABLE 4:** Evidence‐ and expert‐informed RTP protocol tailored to the demands of competitive alpine skiers following ACL injury—Phase 2.

Phase 2: Presurgery	
Timeline: From medical plan confirmation until surgery	
Overall goals	Achieve a “quiet knee”: eliminate all swelling and restore normal walking gait without pain/heat therapy Normalize knee ROM: restore full, symmetrical range of motion, with a primary focus on terminal extension Prepare for surgery: physically and psychologically prepare the athlete for the operation and the subsequent rehabilitation phase; presurgical nutritional intervention Develop a rehabilitation plan and shared decision making
Neuromuscular readiness	Restore ROM: passive ROM (90% of contralateral side) Neuromuscular readiness for surgery: restore muscle function, symmetrical muscle recruitment during squats and leg press (max. 30° flexion) Prevent atrophy and maintain strength in injured and uninjured lower body areas using NMES and BFR Treat arthrogenic muscle inhibition by using controlled isometric contractions and/or NMES (quadriceps & hamstrings) Aquatic therapy Train for hypertrophy and strength in upper‐body areas (including non‐limited trunk exercises)
Psychological & social readiness	Gather information to foster a sense of competence Develop mental toughness with emphasis on resilience, self‐efficacy, perseverance, and self‐awareness Involve mental hardiness with emphasis on commitment, control, and challenge Pain management Seek social support (e.g., peers with injury experience) Promote realistic expectations Identify and reduce stressors The athlete must be and remain communicative and discuss everything that occupies their mind
Perceptual‐motor‐cognitive readiness	Introduce basic neurocognitive training, such as dual‐tasking: e.g., incorporate light cognitive tasks during seated or upper‐body conditioning exercises, or as part of daily routines Introduce reactive drills: introduce simple reactive balance drills (e.g., responding to external perturbations) Introduce movement visualization: practice detailed visualization of ideal, fluid skiing movements and patterns
On‐snow progression	n/a
Monitoring & testing	Monitor ROM/effusion/pain/arthrogenic inhibition using goniometer measurement, the stroke test, knee circumference, and VAS Monitor physical and mental health using the OSTRC‐H2 questionnaire Daily athlete monitoring: sleep, well‐being, heart rate/HRV, activity diary

Abbreviations: BFR, blood flow restriction; HRV, heart rate variability; NMES, neuromuscular electrical stimulation; OSTRC‐H2, Oslo Trauma Research Center questionnaire on health problems—version 2; ROM, range of motion; VAS, visual analogue scale.

**TABLE 5:** Evidence‐ and expert‐informed RTP protocol tailored to the demands of competitive alpine skiers following ACL injury—Phase 3.

Phase 3: Postsurgery	
Timeline: Approximately 0–2 weeks postoperative	
Overall goals	Recover from surgery: physically and mentally Protect the graft: adhere strictly to postoperative protocols to protect the healing graft Manage pain & effusion: diligently control postoperative pain and swelling Restore extension & activate the quads: reestablish full passive extension and achieve voluntary quadriceps activation without an extension lag Normalize gait: progress to safe, normalized gait with crutches as tolerated Postoperative nutritional intervention
Neuromuscular readiness	Progression ROM: passive ROM (extension 100%; flexion 75% of contralateral side) Restore muscle function: symmetrical muscle recruitment during squats and leg press (max. 90° flexion) Prevent atrophy and maintain strength in injured lower body areas using NMES Introduce low volume hypertrophy training of the non‐injured leg focussing on cross‐educational muscle stimulation (progressive introduction of eccentric muscle loading for the quadriceps & hamstrings) Treat arthrogenic muscle inhibition first by reducing swelling, pain, etc., via cryotherapy, local vibration, medication and TENS, followed by modalities that enhance activation by using controlled isometric contractions and/or NMES
Psychological & social readiness	Manage postoperative anxiety: implement strategies for pain management, sleep hygiene, and coping with initial limitations Foster motivation: maintain motivation for fundamental rehab tasks and encourage continued connection with the team Promote self‐compassion: encourage a mindset of patience, cocreate optimistic but realistic mindsets and recovery expectations and mindfulness during the initial recovery period Regain autonomy and self‐efficacy Build and consolidate a supportive environment Find balance and purpose outside of sports The athlete must be and remain communicative and discuss everything that occupies their mind
Perceptual‐motor‐cognitive readiness	Support recovery and cognitive engagement while physical activity is restricted Rebuild perceptual and cognitive readiness without stressing injured tissue Begin perceptual–cognitive–motor training using the uninjured limb Introduce isolated cognitive‐perceptual–motor stimuli Progression: Simple motor plus simple lower‐order cognitive dual tasks
On‐snow progression	n/a
Monitoring & testing	Surgical site integrity monitoring: regularly check the wound status and monitor for any signs of infection Monitor ROM/effusion/pain/arthrogenic inhibition using goniometer measurement, the stroke test, knee circumference, and VAS Monitor physical and mental health using the OSTRC‐H2 questionnaire Daily athlete monitoring: sleep, well‐being, heart rate/HRV, activity diary

Abbreviations: HRV, heart rate variability; NMES, neuromuscular electrical stimulation; OSTRC‐H2, Oslo Trauma Research Center questionnaire on health problems—version 2; ROM, range of motion; TENS, transcutaneous electrical nerve stimulation; VAS, visual analogue scale.

**TABLE 6:** Evidence‐ and expert‐informed RTP protocol tailored to the demands of competitive alpine skiers following ACL injury—Phase 4.

Phase 4: Early rehab	
Timeline: Approximately 2–8 weeks postoperative	
Overall goals	Set the early rehab goals; modulate the rehabilitation plan depending on age, maturity, date of initial injury/surgery with respect to the upcoming competition season Prepare to return‐to‐activity; both physically and mentally Achieve full ROM & normalize gait: attain symmetrical active ROM and normalize gait without assistive devices Maintain effusion control: progress exercises without causing a significant or persistent increase in joint effusion Maintain general fitness: establish an aerobic base and progress quadriceps strength
Neuromuscular readiness	Progress ROM: passive ROM (flexion 100% of contralateral side) Restore unilateral joint function under bodyweight loading Restore movement symmetry Restore neuromuscular control Restore dynamic knee alignment and core stability Restore muscle mass using lower intensity training to muscular failure focussing on achieving the largest possible ROM Introduce muscular strength activities using controlled, non‐load‐dependent movement velocities emphasizing the largest possible ROM Introduce RFD activities using a progressive approach toward rapid isometric muscle contraction Train for hypertrophy and strength in upper‐body areas (including non‐limited trunk exercises)
Psychological & social readiness	Adhere to the rehabilitation plan and adjust it as necessary Build confidence: utilize short‐term goal achievement to build self‐efficacy and manage potential frustration See rehabilitation as athletic performance training Cope with ups and downs; regulate motivation and make progress visible Work on any deficits regarding mental skills for which time was restricted before injury Promote social interaction and integration/maintain team connection Engage in complementary activities and hobbies outside of rehabilitation The athlete must be and remain communicative and discuss everything that occupies their mind
Perceptual‐motor‐cognitive readiness	Focus on simple lower‐order cognitive tasks (e.g., reaction time, attention, tracking) to maintain engagement and prevent cognitive decline Gradual reactivation of the injured limb in a cognitively engaging context Develop safe dual‐task capacity (motor + cognitive) Enhance perceptual awareness and remapping of body schema Transition from uninjured to injured side; simple motor + simple cognitive → simple motor + higher‐order cognitive Progression: controlled physical reintroduction with increased cognitive demand
On‐snow progression	Familiarization with wearing ski boots while training off‐snow– coordinative training with ski boots
Monitoring & testing	Patient‐reported outcomes (PROs): regularly administer scores such as ACL QOL, IKDC, KOOS to track subjective function Morphological assessment: body composition (DXA, anthropometric testing) Functional assessment: assess dynamic balance (e.g., Y‐Balance Test) perform qualitative gait symmetry analysis Strength assessment: quantify quadriceps and hamstring strength LSI using isokinetic or handheld dynamometry Cardiovascular fitness assessment: incremental bike test Psychological assessment: assess mental health using the SMHAT‐1 or a similar tool Monitor load response: monitor effusion levels 24 h after introducing new or more intense exercises Monitor physical and mental health using the OSTRC‐H2 questionnaire Daily athlete monitoring: sleep, well‐being, heart rate/HRV, activity diary, internal training load (e.g., sessional RPE)

Abbreviations: ACL QOL, anterior cruciate ligament quality of life questionnaire; DXA, dual‐energy X‐ray absorptiometry; HRV, heart rate variability; IKDC, international knee documentation committee score; KOOS, knee injury and osteoarthritis outcome score; LSI, limb symmetry index; OSTRC‐H2, Oslo Trauma Research Center questionnaire on health problems—version 2; PRO, patient‐reported outcomes; RFD, rate of force development; ROM, range of motion; RPE, rate of perceived exertion; SMHAT‐1, sport mental health assessment tool; VAS, visual analogue scale.

**TABLE 7:** Evidence‐ and expert‐informed RTP protocol tailored to the demands of competitive alpine skiers following ACL injury—Phase 5.

Phase 5: Mid rehab	
Timeline: Approximately 2–4 months postoperative	
Overall goals	Set the mid‐rehab goals; modulate the rehabilitation plan depending on age, maturity, date of initial injury/surgery with respect to the upcoming competition season Prepare to return‐to‐snow; both physically and mentally Achieve strength targets: reach key strength milestones; develop power foundations: introduce and progress plyometric and change‐of‐direction drills Introduce sport‐specific loading: begin incorporating movements that mimic the demands of skiing Build on‐snow confidence: initiate a highly controlled, criteria‐led return to snow focused on fundamental skills
Neuromuscular readiness	Restore muscle strength and maximal load tolerance through unilateral‐ and bilateral movements in full ROM; progress movement symmetry to more advanced motor skills (e.g., running drills) Progress neuromuscular control; progress dynamic knee alignment by incorporating preparatory landing control exercises and low‐intensity weightlifting catching derivatives Restore muscle mass using moderate intensity training primarily to muscular failure emphasizing full ROM (increase time under tension during the eccentric phase) Restore slow supramaximal eccentric loading of the hamstring muscles, focussing on long muscle‐tendon lengths (knee‐ and hip‐dominant exercises) Progress muscular strength activities using augmented load dependent movement velocities; progress RFD activities toward rapid dynamic concentric muscle contraction and the integration of weightlifting pulling derivatives Progress core stability and strength through the use of strength‐endurance and heavy‐load exercises; train for hypertrophy and strength in upper‐body areas
Psychological & social readiness	Adhere to the rehabilitation plan and adjust it as necessary Build confidence: utilize short‐term goal achievement to build self‐efficacy and manage potential frustration See rehabilitation as athletic performance training Cope with ups and downs; regulate motivation and make progress visible Evaluate and treat kinesiophobia, fear and anxiety of reinjury and/performance drop Work on any deficits regarding mental skills for which time was restricted before injury Occasional contact with regular teammates and coaches The athlete must be and remain communicative and discuss everything that occupies their mind
Perceptual‐motor‐cognitive readiness	Focus on dual‐task training, combining motor work with cognitive challenges of varying complexity to rebuild cognitive–motor coordination and adaptability Reinforce cognitive–motor integration under increasing physical load; increase task complexity through variable environments and higher cognitive demands (off‐snow) Rebuild perceptual decision‐making related to dynamic skiing movements, e.g., by video‐based tactical decision‐making training; consider VR simulations if available (off‐snow) Improve the ability to adequately react to external perturbations (visual, acoustic, sensory, and vestibular) during controlled movement tasks (off‐snow) Improve skiers ability to sense snow conditions, boot and ski pressure, body posture (on‐snow)
On‐snow progression	First on‐snow sessions (appropriate physical and psychological recovery level provided!); clearance for on‐snow skiing informed by the orthopedic surgeon and based on interprofessional shared decision making; start with short session—increase session length slowly Restricted rehabilitation team‐led skiing: free skiing starting with the absolute basics, low speeds and skidded (not carved turns) Progressively building up in terms of various parameters (e.g., forms of exercise, speed, equipment, slope conditions, terrain, visibility, etc.) Progressively implementing carved turns on the snow carpet (tissue tolerance to more specific and complex loading) Bridge the gap between neuromuscular deficits in the clinic to the hill
Monitoring & testing	Patient‐reported outcomes (PROs): regularly administer scores such as IKDC or KOOS to track subjective function Morphological assessment: body composition (DXA, anthropometric testing) Functional assessment: use video analysis to assess movement quality during key exercises (e.g., squats, step‐downs, drop jumps) Strength assessment: isokinetic knee extensor/knee flexor/leg extensor strength testing Cardiovascular fitness assessment: incremental bike test Psychological assessment: assess mental health using the SMHAT‐1 or a similar tool Monitor load response: monitor effusion levels 24 h after introducing new or more intense exercises Monitor power capacities and asymmetries: weekly CMJ force assessment Monitor physical and mental health using the OSTRC‐H2 questionnaire Daily athlete monitoring: sleep, well‐being, heart rate/HRV, activity diary, internal training load (e.g., sessional RPE)

Abbreviations: CMJ, countermovement jump; DXA, dual‐energy X‐ray absorptiometry; HRV, heart rate variability; IKDC, international knee documentation committee score; KOOS, knee injury and osteoarthritis outcome score; OSTRC‐H2, Oslo Trauma Research Center questionnaire on health problems—version 2; PRO, patient‐reported outcomes; RFD, rate of force development; ROM, range of motion; RPE, rate of perceived exertion; SMHAT‐1, sport mental health assessment tool; VAS, visual analogue scale; VR, virtual reality.

**TABLE 8:** Evidence‐ and expert‐informed RTP protocol tailored to the demands of competitive alpine skiers following ACL injury—Phase 6.

Phase 6: Late rehab	
Timeline: Approximately 4–6+ months postoperative	
Overall goals	Set the late rehab goals; modulate the rehabilitation plan depending on age, maturity, date of initial injury/surgery with respect to the upcoming competition season Prepare to return‐to‐sport; both physically and mentally Work on the remaining physical and mental weaknesses identified (advise the athlete sufficient time to eliminate all of them before returning to sport) Maximize neuromuscular performance: optimize strength, power, and fatigue resistance to meet sport‐specific demands Refine ski‐specific skills: develop tolerance for complex, multi‐planar movements on varied terrain and snow conditions Prepare for team training: satisfy all objective and subjective criteria required to reintegrate into early team training Develop a plan for the early and regular team training phases (with flexibility); consult/educate the athlete; The decision to return to sport should be informed by the orthopedic surgeon and should be based on interprofessional shared decision‐making
Neuromuscular readiness	Optimize overall athletic performance and robustness with focus on maximizing concentric, isometric and eccentric strength and power Restore the intensity, specificity and complexity of the motion tasks to their preinjury levels Optimize task‐specific muscle mass and muscular strength using high‐intensity training emphasizing maximal movement intent during the concentric phase Optimize slow supramaximal eccentric loading of the quadriceps and hamstring muscles, focusing on long muscle‐tendon lengths and a gradual integration of tibial rotation movement to the latter Optimize RFD activities through plyometric training emphasizing slow SSC and the integration of moderate intensity weightlifting catching derivatives as well as sprint drills Full recovery of movement symmetry through progressive exposure to multidirectional movements Train for hypertrophy and strength in upper‐body areas
Psychological & social readiness	Adhere to the rehabilitation plan and adjust it as necessary See rehabilitation as athletic performance training Cope with ups and downs; regulate motivation and make progress visible Rebuild self‐confidence and foster resilience Work on any deficits regarding mental skills for which time was restricted before injury Occasional visits to regular team training sessions Early identification of psychological red flags that need a more flexible timeframe The athlete must be and remain communicative and discuss everything that occupies their mind
Perceptual‐motor‐cognitive readiness	Focus on complex perceptual–motor tasks with alternating cognitive demands — both low (automatic execution under fatigue) and high (strategic decision‐making)—to ensure performance stability under real‐world variability Improve motor‐cognitive dual task capacity during complex movement tasks (off‐snow) Improve the capacity to adapt to varying demands of eccentric muscle loading, both horizontal axial loading (off‐snow) Improve dynamic knee alignment and core stability through perturbation training, explosive anti‐rotation exercises and heavy‐load exercises during squats, deadlift, and hip thrust (off‐snow) Restriction of perception of one or more visual, acoustic, sensory, and vestibular channels while skiing (on‐snow)
On‐snow progression	Unrestricted coach‐led skiing: progress from supervised or modified off‐snow training to unsupervised or unmodified on‐snow activity (approaching full speed and carved turns) Introduce gates: begin with easy courses (e.g., stubbies, sparse full gates) on moderate terrain with low speeds The back‐to‐sport decision encompasses the ability to engage in “normal skiing” with typical speeds and forces
Monitoring & testing	Patient‐reported outcomes (PROs): regularly administer scores such as IKDC or KOOS to track subjective function Morphological assessment: body composition (DXA, anthropometric testing) Functional assessment: use 3D motion capture to assess movement quality during key exercises (e.g., squats, drop jumps) Strength assessment: isokinetic knee extensor/knee flexor/leg extensor strength testing Cardiovascular fitness assessment: incremental bike test Psychological assessment: assess mental health using the SMHAT‐1 or a similar tool; measure psychological readiness using the ACL‐RSI scale Monitor load response: monitor effusion levels 24 h after introducing new or more intense exercises Monitor power capacities and asymmetries: weekly CMJ force assessment Monitor physical and mental health using the OSTRC‐H2 questionnaire Daily athlete monitoring: sleep, well‐being, heart rate/HRV, activity diary, internal training load (e.g., sessional RPE), external training load (e.g., run count, acceleration load with IMU)

Abbreviations: CMJ, countermovement jump; DXA, dual‐energy X‐ray absorptiometry; HRV, heart rate variability; IKDC, international knee documentation committee score; IMU, inertial measurement unit; KOOS, knee injury and osteoarthritis outcome score; OSTRC‐H2, Oslo Trauma Research Center questionnaire on health problems—version 2; PRO, patient‐reported outcomes; RFD, rate of force development; ROM, range of motion; RPE, rate of perceived exertion; SMHAT‐1, sport mental health assessment tool; SSC, stretch‐shortening cycle; VAS, visual analogue scale.

**TABLE 9:** Evidence‐ and expert‐informed RTP protocol tailored to the demands of competitive alpine skiers following ACL injury—Phase 7.

Phase 7: Early team training	
Timeline: Approximately 9–12+ months postoperative	
Overall goals	Set the early team training goals; shift focus from rehabilitation milestones to performance and career goals Prepare to return‐to‐competition; both physically and mentally Achieve competition readiness: progress overall physical, mental, technical and tactical off‐ and on‐snow performance to 100% of the preinjury level or beyond Manage overall load: effectively balance on‐snow training, conditioning, travel, and recovery to prevent overload Structured reintegration: safely and effectively reintegrate into the full team training environment Regain competitive form: build confidence and tactical sharpness within a group and simulated competitive setting; clearance for competition should be based on interprofessional shared decision‐making
Neuromuscular readiness	Optimize overall athletic performance and robustness with focus on maximizing concentric, isometric and eccentric strength and power by preparing for accentuated eccentric loading Progress the preinjury intensity, specificity and complexity of the motion tasks Optimize task‐specific muscle mass and muscular strength using high‐intensity training emphasizing maximal movement intent during the concentric phase Intensify eccentric loading of the hamstring muscles using fast eccentric loading Intensify RFD activities through plyometric training emphasizing short GCT, the use of weightlifting derivatives and the integration of reflexive eccentrics as well as multidirectional sprint drills Train for strength and power in upper‐body areas
Psychological & social readiness	Ensure complete information transfer from the rehabilitation to the training setting Build competitive confidence: reestablish confidence in a competitive environment by managing performance anxiety and resuming individually appropriate risk‐taking (risk perception evaluation with subjective/objective factors) Reestablish team role: getting back to the regular team, settle back into team dynamics, and on‐hill communication routines The athlete must be and remain communicative and discuss everything that occupies their mind
Perceptual‐motor‐cognitive readiness	Restore full perceptual–motor integration and reactivity (off‐snow) Train rapid perception–decision–action loops under controlled conditions (off‐snow) Address fear and hesitancy with confidence‐building cognitive drills(off‐snow) Optimize the capacity to adapt to varying demands of eccentric muscle loading/multidirectional loading (off‐snow) Maximize dynamic knee alignment and core stability through perturbation training, explosive anti‐rotation exercises and heavy‐load exercises during squat, deadlifts, and hip thrust (off‐snow) Improve perception of various external objects (other skiers, obstacles, gates, terrain changes, etc.) when skiing at high speed and with limited time for planning (on‐snow) Improve the reactive control of unexpected and highly dynamic forces resulting from the equipment's self‐steering‐behavior during the ski‐snow interaction (on‐snow)
On‐snow progression	Variations as typical for competitive skiing: on‐snow training under different conditions of snow, visibility, terrain, course set, equipment; variations in timing, intensity, rhythm, and overall skiing volume; variations between free‐skiing and in gates Full‐speed tactical execution training: engage in training that requires real‐time reactions to teammates and changing environments Race simulation: complete full race simulations, from course inspection and warm‐up to timed runs and postrace analysis The back‐to‐competition decision encompasses the ability to engage in competitive skiing and resume risk‐taking
Monitoring & testing	Biannual morphological assessment: body composition (DXA, anthropometric testing) Biannual neuromuscular performance testing: lower limb eccentric power, COD, agility and RFD Biannual fitness performance testing: maximal aerobic power, critical power, anaerobic capacity Biannual psychological assessment: assess mental health using the SMHAT‐1 or a similar tool Monitor power capacities and asymmetries: weekly CMJ force assessment Monitor physical and mental health using the OSTRC‐H2 questionnaire Daily athlete monitoring: sleep, well‐being, heart rate/HRV, activity diary, internal training load (e.g., sessional RPE), external training load (e.g., run count, acceleration load with IMU) On‐snow load and biomechanical monitoring: use GPS/IMU/CV to monitor on‐snow volume, intensity, and turn symmetry

Abbreviations: CMJ, countermovement jump; COD, change of direction; CV, computer vision; DXA, dual‐energy X‐ray absorptiometry; GCT, ground contact time; GPS, global position system; HRV, heart rate variability; IMU, inertial measurement unit; OSTRC‐H2, Oslo Trauma Research Center questionnaire on health problems—version 2; RFD, rate of force development; RPE, rate of perceived exertion; SMHAT‐1, sport mental health assessment tool.

**TABLE 10:** Evidence‐ and expert‐informed RTP protocol tailored to the demands of competitive alpine skiers following ACL injury—Phase 8.

Phase 8: Regular team training	
Timeline: Ongoing throughout the athlete's career	
Overall goals	Set the short ‐, mid‐, and long‐term performance goals Prepare to return‐to‐performance; both physically and mentally Optimize performance: improve overall physical, mental technical and tactical off‐ and on‐snow performance and achieve or exceed preinjury performance levels Embed injury prevention: integrate secondary injury prevention strategies into the athlete's routine Maximize career longevity: optimize long‐term athletic development and health Push performance limits: continuously work to enhance all aspects of physical, mental, technical, and tactical performance Offload the athlete with inadequate pressure
Neuromuscular readiness	Maximize overall athletic performance and robustness with focus on maximizing concentric, isometric and eccentric strength and power by using AEL Maximize the preinjury intensity, specificity and complexity of the motion tasks Maximize task‐specific muscle mass and muscular strength using high‐intensity training while emphasizing maximal movement intent during the concentric phase Maximize eccentric loading of the hamstring muscles using impulse loading Maximize RFD activities through plyometric training focusing on short GCT and AEL, heavy loaded weightlifting derivatives and intensified reflexive eccentrics and multidirectional sprint drills Train for strength and power in upper‐body areas including the core
Psychological & social readiness	Performance‐focused mental skill training Cope with elite sport demands: effectively manage the stress, travel, and lifestyle demands of a professional athlete Intuitive decision‐making: make rapid, effective tactical decisions at world‐class race speeds Foster resilience: develop robust coping mechanisms to handle performance setbacks and successes The athlete must be and remain communicative and discuss everything that occupies their mind
Perceptual‐motor‐cognitive readiness	Dual‐task mastery: seamlessly integrate internal feedback (body position) and external cues (course, competitors) during motor performance tasks (off‐snow) Improve motor‐cognitive dual task capacity during complex movement tasks (on‐snow) High‐level adaptability: demonstrate expert ability to adapt to unpredictable race‐day conditions (on‐snow)
On‐snow progression	Full competition and training load: follow a complete competition and training schedule dictated by seasonal goals Return to the slope/course of the accident; overcoming remaining doubts and fears, finding peace and moving on
Monitoring & testing	Biannual morphological assessment: body composition (DXA, anthropometric testing) Biannual neuromuscular performance testing: lower limb eccentric power, COD, agility and RFD Biannual fitness performance testing: maximal aerobic power, critical power, anaerobic capacity Biannual psychological assessment: assess mental health using the SMHAT‐1 or a similar tool Monitor power capacities and asymmetries: weekly CMJ force assessment Monitor physical and mental health using the OSTRC‐H2 questionnaire Daily athlete monitoring: sleep, well‐being, heart rate/HRV, activity diary, internal training load (e.g., sessional RPE), external training load (e.g., run count, acceleration load with IMU) On‐snow load and biomechanical monitoring: use GPS/IMU/CV to monitor on‐snow volume, intensity, and turn symmetry Long‐term analytics: analyze long‐term load and performance data to optimize training and minimize injury risk

Abbreviations: AEL, accentuated eccentric loading; CMJ, countermovement jump; COD, change of direction; CV, computer vision; DXA, dual‐energy X‐ray absorptiometry; GCT, ground contact time; GPS, global position system; HRV, heart rate variability; IMU, inertial measurement unit; OSTRC‐H2, Oslo Trauma Research Center questionnaire on health problems—version 2; RFD, rate of force development; RPE, rate of perceived exertion; SMHAT‐1, sport mental health assessment tool.

### 4.4 Methodological Considerations

Despite the comprehensive search strategy, robust study selection, and data extraction and synthesis methodology, several limitations should be considered when the study findings are interpreted.

First, owing to the limited amount of evidence on RTS/RTP following ACL injury in competitive alpine skiing, this study was designed as a scoping review to broadly explore the available literature rather than to address a specific research question and was supplemented by the clinical/practical expertise input of the authors. The resulting recommendations therefore offer evidence‐ and expert‐informed practice recommendations rather than definitive standards. This may have introduced certain subjectivity, which should be resolved through further studies. One option would be to strive for a consensus with a larger panel of international experts from a range of disciplines, as well as clinicians and patients, to formulate widely applicable recommendations for “best practices.” Second, owing to the complex nature of sports injury rehabilitation and its associated research, publication bias may be a limitation of this study. To counteract this limitation, particular emphasis was also given to creative activities among the author group, in which the authors' extensive practical experience was used to develop practice guidelines. Third, the included studies are at risk of selection bias. To counteract this risk, different databases relevant to the topic were used for the literature search. Moreover, a professional librarian optimized the search string using a search validation procedure based on preselected key articles, and a two‐assessor strategy was employed to increase the reliability of the study selection procedure. Fourth, for efficiency reasons, data extraction was essentially performed by one assessor only. However, to ensure reliability and completeness, a second assessor extracted the data from the first five articles in parallel, and the results were compared. Fifth, given the small number of publications in this field, there is a high risk of overlapping authorship. Any potential bias resulting from this was addressed by two independent assessors, who screened the studies for eligibility. If both assessors were authors of a study that was included, a third independent assessor (selected on a case‐by‐case basis) reassessed its eligibility.

## 5 Conclusions

This scoping review provides a comprehensive overview of the current literature on RTS/RTP following ACL injury in competitive alpine skiing and offers novel insights into aspects of practical implementation. Our findings advocate for a multi‐domain, criterion‐based RTS/RTP framework for ACL injury recovery, emphasizing neuromuscular, psycho‐social, and perceptual–motor–cognitive readiness, alongside social support, on‐snow progression, and continuous monitoring/testing. To bridge the remaining knowledge gaps, we present a comprehensive, phased approach that considers physical, mental, and performance factors. This is in the form of an evidence‐ and expert‐informed RTP protocol for competitive alpine skiers after ACL injury. The protocol was developed by the authors alongside a collection of practical ideas and illustrative reports of clinical and practical experience. These documents are intended to provide evidence‐ and expert‐based recommendations for practice.

## 6 Perspectives

Most studies on ACL injury have focused on epidemiology, secondary injury risk, and early rehabilitation. However, evidence regarding later recovery phases following return to activity (i.e., return to snow, return to sport, return to competition and return to performance) remains limited, and little evidence on the psychological and social factors influencing RTS/RTP exists. Future research is needed to address these knowledge gaps and develop evidence and expert‐informed practices that are based on stronger evidence. Moreover, whether the present evidence and expert‐informed practices for competitive alpine skiers can also be transferred to competitive para alpine skiers or recreational skiers remains to be verified by further research dedicated to these particular populations.

## Author Contributions

J.Sp. and H.‐C.H. were responsible for the conception of the study. J.Sp. planned and coordinated the literature search. J.Sp. and P.O.M. independently assessed the records. M.J.J. acted as the third assessor if there was any disagreement between J.Sp. and P.O.M. J.Sp. and P.O.M. extracted the data with the supervision of H.H. J.Sp. synthesized the data; all the authors critically reviewed the synthesis. J.Sp. and H.‐C.H. were responsible for the first draft of the manuscript. All the authors contributed to the interpretation of the findings, content generation, and critical revision of the manuscript and reviewed and approved the final manuscript.

## Funding

The authors have nothing to report.

## Ethics Statement

The authors have nothing to report.

## Conflicts of Interest

The authors declare no conflicts of interest.

## Supporting information


**File S1:** sms70246‐sup‐0001‐FileS1.docx.

**File S2:** sms70246‐sup‐0002‐FileS2.docx.

**File S3:** sms70246‐sup‐0003‐FileS3.docx.

## Data Availability

All relevant data generated or analyzed during this study are included in this published article and its Supporting Information files.
