# Supplementary material for: Returning to Performance After ACL Injury in Competitive Alpine Skiing: A Scoping Review and Evidence‐ and Expert‐Informed Practice Recommendations
Source: Scand J Med Sci Sports. 2026 Mar 8;36(3):e70246. doi: 10.1111/sms.70246 (PMC12968495; doi:10.1111/sms.70246)
Supplement: Supplementary file 1 — File S1: sms70246‐sup‐0001‐FileS1.docx. [file SMS-36-e70246-s001.docx]

**Supplemental File 1: Overview of the extracted data**

***PART 1: study characteristics such as general study descriptors, injury patterns and surgical details***

| **Author/Year** | **General Study Descriptors** | **Injury Patterns** | **Surgical Details** |
| --- | --- | --- | --- |
| **Bianco (2001)** | Qualitative study; competitive alpine skiers (n = 10) | Severe injury including ACL injury | n/a |
| **Bianco et al. (1999)** | Qualitative study; competitive alpine skiers (n = 12) | Severe injury including ACL injury | n/a |
| **Brucker et al. (2016)** | Clinical best practice description for competitive alpine skiers | ACL injury and reconstruction | n/a |
| **Csapo et al. (2019)** | Retrospective observational study; competitive alpine skiers (n = 46) | ACL injury and reconstruction; both simple ACL injuries or complex ACL injuries (multiligament injuries or involvement of menisci or cartilage) | Hamstring grafts and quadriceps tendon graft |
| **Csapo et al. (2021)** | Retrospective observational study; competitive alpine skiers (n = 30) | Secondary ACL reinjury | Hamstring grafts and quadriceps tendon graft |
| **DePhillipo et al. (2020)** | Case report; female Olympic alpine skier (n = 1) | ACL and LCL injury accompanied by a complex radial tear of the lateral meniscus, medial meniscus tear, popliteofibular ligament tear, proximal tibiofibular joint ligament tear, and a common peroneal nerve neuropraxia | Anatomic single-stage, multi-ligament knee reconstruction surgery including an ACL reconstruction with patellar tendon autograft and a novel meniscus radial repair technique |
| **Ekeland et al. (2020)** | Retrospective observational study; athletes (n = 14201); alpine skiers (n = 2693; including recreational and competitive skiers), football players (n = 8498) and handball players (n = 3010) | Secondary ACL reinjury | Hamstring grafts and patellar tendon grafts |
| **Farinelli et al. (2023)** | Retrospective observational study; athletes n=81; competitive soccer players (n = 37) and competitive alpine skiers (n = 44) | ACL injury and reconstruction; both simple ACL injuries or complex ACL injuries (multiligament injuries or involvement of menisci or cartilage) | n/a |
| **Guy et al. (2022)** | Retrospective observational study; competitive alpine skiers (n = 59) | Secondary ACL reinjury | ACL reconstruction (hamstring grafts and patellar tendon grafts) or combined procedures (ACL reconstruction + LEAP) |
| **Haida et al. (2016)** | Prospective observational study; competitive alpine skiers (n = 477) | Skiers with ACL injury and reconstruction, and skiers without | n/a |
| **Jedvaj et al. (2021)** | Cross-sectional study; competitive alpine skiers (n = 33) | Severe injury including ACL injury | n/a |
| **Jordan et al. (2015a)** | Cross-sectional study; competitive alpine skiers (n = 18) | Skiers with ACL injury and reconstruction, and skiers without | n/a |
| **Jordan et al. (2015b)** | Cross-sectional study; competitive alpine skiers (n = 29) | Skiers with ACL injury and reconstruction, and skiers without | Hamstring graft and allograft |
| **Jordan et al. (2017a)** | Cross-sectional study; competitive alpine skiers (n = 22) | Skiers with ACL injury and reconstruction, and skiers without | Hamstring graft, patellar tendon graft and allograft |
| **Jordan et al. (2017b)** | Retrospective observational study; competitive alpine skiers (n = 39) | ACL injury and reconstruction | Hamstring graft, patellar tendon graft, quadriceps tendon graft, allograft/autograft hybrid and allograft |
| **Jordan et al. (2018)** | Cross-sectional study; competitive alpine skiers (n = 36) | Skiers with ACL injury and reconstruction, and skiers without | n/a |
| **Jordan et al. (2020)** | Case study (n=1); female competitive alpine skier | High grade ACL tear, medial meniscal tear, lateral tibial plateau compression fracture | ACL reconstruction surgery using an 8.5 mm quadruple semitendinosus autograft |
| **Jordan et al. (2022b)** | Cross-sectional study; female competitive alpine skiers and ski cross athletes (n = 47) | Skiers with ACL injury and reconstruction, and skiers without | Semitendinosus tendon autograft |
| **Kokmeyer et al. (2012)** | Clinical best practice description for competitive alpine skiers | ACL injury and reconstruction | n/a |
| **Morris et al. (2025)** | Retrospective observational study; competitive alpine skiers (n = 30) | ACL injury and reconstruction; both simple ACL injuries or complex ACL injuries (multiligament injuries or involvement of menisci or cartilage) | Hamstring tendon autograft, bone–patellar tendon–bone autograft, quadriceps tendon autograft, allograft |
| **Müller et al. (2023)** | Clinical best practice description for competitive snow sports athletes including alpine skiers | Severe injury including ACL injury | n/a |
| **Müller et al. (2024)** | Qualitative study; expert stakeholders in competitive snow sports (n=14) | Severe injury including ACL injury | n/a |
| **Nordahl et al. (2014)** | Qualitative study; competitive alpine skiers (n=5) | ACL injury and reconstruction | n/a |
| **Nwachukwu et al. (2017)** | Retrospective observational study; competitive athletes (n=294); soccer players (n=92); competitive; alpine skiers (n=67), basketball players (n=56); lacrosse players (n=38), football players (n=29); tennis players (n=12) | ACL injury and reconstruction | n/a |
| **Praz et al. (2019)** | Case report (n=1); female Olympic alpine skier | ACL injury and reconstruction; injury and reconstruction of the ALL | ACL repair and an ALL-reconstruction using semitendinosus allograft |
| **Pujol et al. (2019)** | Prospective observational study; competitive alpine skiers (n=379) | Skiers with ACL injury and reconstruction, and skiers without | n/a |
| **Rosenstiel et al. (2019)** | Retrospective observational study; competitive athletes (n=70); soccer players (n=32); rugby players (n=11); basketball players (n=10); alpine skiers (n=9); handball players (n=4); hockey players (n=2); motocross (n=2) | ACL injury and reconstruction; injury and reconstruction of ALL | Semitendinosus tendon and gracilis tendon graft |
| **Smith et al. (2024)** | Clinical best practice description for competitive alpine skiers | ACL injury and reconstruction | n/a |
| **Spiess et al. (2019)** | Case-control study; competitive alpine skiers (n=88) | Skiers with ACL injury and reconstruction, and skiers without | n/a |
| **Stevenson et al. (1998)** | Retrospective observational study; competitive alpine skiers (n=440) | Skiers with ACL injury and reconstruction, and skiers without | n/a |
| **Strobel et al. (2001)** | Case report (n=1); female 16-year-old competitive alpine skier | Partial ACL rupture and reconstruction: post-surgery: painful reflex extension loss due to femoral malplacement of the ACL graft | Semitendinosus tendon graft |
| **Tecklenburg et al. (2007)** | Case report (n=1); female 19-year-old competitive alpine skier | ACL injury with simultaneous locked bucket-handle tears of both medial and lateral meniscus | ACL: semitendinosus-gracilis graft; medial meniscus: The displaced bucket-handle was reduced and repaired by using six inside-out sutures as well as one all-inside FasT-Fix TM suture on the posterior horn of the meniscus; lateral meniscus: The bucket-handle was reduced and repaired with four FasT-Fix TM sutures |
| **Udry et al. (1997)** | Qualitative study; competitive alpine skiers (n = 21) | Severe injury including ACL injury | n/a |
| **Verdonk et al. (2024)** | Case report (n=1); young Olympic alpine skier | ACL injury accompanied by a fracture of the posteromedial tibial plateau, detachment of the medial collateral ligament and tears of both menisci | Seven surgical procedures including ACL reconstruction using an autologous patellar tendon graft |
| **Westin et al. (2022)** | Prospective observational study; adolescent competitive alpine skiers (n=31) | Secondary ACL reinjury | n/a |

ACL: anterior cruciate ligament; LEAP: lateral extra-articular procedure; ALL: anterolateral ligament; FasT-Fix TM: specific type of meniscal repair system.

***PART 2: rehabilitation/training activities, testing/monitoring metrics and key outcomes***

| **Author/Year** | **Rehabilitation/Training** | **Testing/Monitoring** | **Key Outcomes** |
| --- | --- | --- | --- |
| **Bianco (2001)** | n/a | n/a | - Emotional, informational, and tangible support is key - Potential sources of support: treatment team, ski team, and home support networks - Support is important for reducing distress and maintaining motivation during recovery |
| **Bianco et al. (1999)** | n/a | n/a | - Each RTS/RTP phase is marked by a series of events that causes the skiers varying degrees of distress - Strategies to manage stress are key |
| **Brucker et al. (2016)** | Return-to-activity   - Anti-inflammatory, analgesic, and wound-healing measures - Exercises for (neuro)muscular control of the thigh muscles   Return-to-sport   - Increase of mobility and strength, considering the partial/full load - Therapeutic skiing & sensory-motor exercises on skis   Return-to-competition   - Back to maximal off-snow training and competitive ski training | Return-to-activity   - Isokinetic strength testing - ROM during squat test - ROM during Y-balance test   Return-to-sport   - Isokinetic strength testing - Lateral step-down-test   Return-to-competition   - Isokinetic strength testing - Drop jump test - Single leg hop test - Side-to-side test | - Best practice recommendations for a multimodal RTS/RTP protocol including an on-snow progression |
| **Csapo et al. (2019)** | n/a | Comprehensive set of fitness data reflecting muscle strength and power (161.5 ± 24.2 days after ACL reconstruction)  Patient reported outcomes (3 months, 5 months, 12 months, 24 months)  FIS points reflecting the athletes’ level of performance prior to injury as well as after return to competition | - Persistent muscle strength and power deficits 161.5 ± 24.2 days after surgery - More pronounced strength and power deficits in quadriceps tendon graft reconstructed skiers than in those with hamstring tendon grafts - FIS points increased after return to competition (364.3 ± 142.5 days post-reconstruction) but dropped below pre-injury levels within the first year |
| **Csapo et al. (2021)** | n/a | n/a | - 46.7% suffered secondary ACL injuries on average 29.4 ± 22.5 months after primary reconstruction - Subsequent contralateral ACL injuries being more common than graft failures (30% vs. 16.7%) - By tendency greater odds of contralateral ACL tears in skiers with hamstring tendon grafts or those with simple primary ACL injuries |
| **DePhillipo et al. (2020)** | Non-weight bearing for six weeks with limited knee flexion ROM of 0 to 90 degrees for two weeks  Stationary cycling after 7 weeks, followed by a generalized strengthening program which focused on muscular endurance  Blood flow restriction training three months postoperatively  Return-to-snow training seven postoperatively  Return to competition 12 months postoperatively  Return-to-performance 14 months postoperatively | Vail sports test at 10 months postoperatively for evaluating RTS clearance | - An athlete can basically return to elite level of competition following an anatomic single-stage, multi-ligament knee reconstruction with a novel meniscus radial repair in a safe but timely manner - Best practice recommendations for a multimodal RTS/RTP protocol including an on-snow progression |
| **Ekeland et al. (2020)** | n/a | n/a | - In alpine skiers (including recreational and competitive skiers), 3.8% of the primary ACL reconstructions were revised within the subsequent 6 years - 5 times higher hazard ratios for graft revision in individuals aged ≤18 years than for those aged ≥35 years (non-skiing specific result) - 1.8 times higher hazard ratio for graft revision in individuals with hamstring grafts that hose with patellar tendon grafts (non-skiing specific result) |
| **Farinelli et al. (2023)** | n/a | n/a | - 68% of the ACL reconstructed competitive alpine skiers showed concomitant meniscal injuries - 34% of the ACL reconstructed competitive alpine skiers showed concomitant chondral injuries |
| **Guy et al. (2022)** | n/a | n/a | - Higher graft rupture rates in isolated ACL reconstructed skiers (34%) than in skiers with ACL reconstruction + LEAP (6.5%) |
| **Haida et al. (2016)** | n/a | n/a | - It is basically possible to return to preinjury or even higher levels of performance after an ACL injury - Mean age at ACL injury was found to be 22.6 ± 4.1 years for male skiers and 19.9 ± 3.5 years for female skiers - Skiers who improved their performances after ACL rupture were significantly younger at the time of injury than those showing a performance deterioration after ACL rupture |
| **Jedvaj et al. (2021)** | n/a | n/a | - Skiers suffered on average form 2.45 knee injuries during their career, most commonly involving the ACL and meniscus - A high level of kinesiophobia was found in 36% of skiers, whereas there were no differences between the sexes - Older skiers were found to have lower fear of recurrent injuries |
| **Jordan et al. (2015a)** | n/a | Functional asymmetry in the countermovement jump, squat jump, and leg muscle mass | - Compared with non-injured skiers, ACL reconstructed skiers revealed having increased asymmetry in muscle mass , kinetic impulse in the countermovement jump concentric phase, and the final phase of the squat jump |
| **Jordan et al. (2015b)** | n/a | Isokinetic dynamometry; maximal torque and rate of torque development | - ACL-reconstructed limbs demonstrated significant maximal torque and rate of torque development (late phase) deficits in the hamstrings and quadriceps muscles compared with the contralateral limb - Increased hamstring/quadriceps ratio in the rate of torque development (first 50 ms) in ACL-reconstructed skiers compared with that in uninjured controls, suggesting enhanced ACL protection - Greater hamstring/quadriceps ratio in the rate of torque development (first 50ms) in non-injured females than in non-injured males, suggesting enhanced ACL protection |
| **Jordan et al. (2017a)** | n/a | 80-s repeated squat jump test on a dual force plate system with simultaneous EMG recordings in vastus lateralis, vastus medialis, semitendinosus, and biceps femoris | - ACL reconstructed athletes displayed a systematic change in asymmetry in the late phase of the jump take-off - Compared with the non-injured limb, the affected limb of ACL reconstructed skiers showed lower quadriceps EMG activity at take-off, prelanding, and postlanding along with increased hamstring activity prelanding and postlanding |
| **Jordan et al. (2017b)** | n/a | n/a | - A majority of the ACL reconstructed knees (82%) demonstrated concurrent injury: 32% multiligament injuries; 29% ipsilateral medial collateral ligament injuries; 54% chondral lesions (majority in the lateral knee compartment); and 82% meniscal tears - Bilateral ACL tears were seen in 22% of the injured skiers - 28% of the injured skiers underwent ACL revision surgery |
| **Jordan et al. (2018)** | n/a | Maximal countermovement jumps and squat jumps with simultaneous ground reaction force measurement using a dual force plate | - Adolescent skiers demonstrated decreased eccentric deceleration impulse and systematic right limb dominance in lower limb stiffness compared with ACL reconstructed and elite skiers |
| **Jordan et al. (2020)** | Multi-faceted return to sport training plan delivered by an interdisciplinary performance team including bio-psycho-social dimensions | Workload monitoring; dual force plate vertical jump force-time assessments (countermovement jumps and squat jumps); 80s repeated squat jump test; maximal voluntary contractions of isometric leg press, knee extension and knee flexion | - While the athlete returned to snow 7 months post-injury, presenting with interlimb asymmetries below 10%, functional and strength deficits persisted up to 18 months post-injury |
| **Jordan et al. (2022b)** | n/a | Countermovement jumps on a dual force plate system | - Between-limb asymmetry decreased in ACL reconstructed skiers with time-from-surgery to reach non-injured control values by 2 years, but stretch-shorten-cycle function, such as maximal vertical jump height and peak external mechanical power remained depressed up to 5 years post-surgery |
| **Kokmeyer et al. (2012)** | Multi-faceted return to sport training plan with focus on physical aspects including an on-snow progression | Qualitative clinical and functional criteria, isokinetic strength | - Example program directed toward safely returning the athlete to alpine skiing following ACL injury |
| **Morris et al. (2025)** | n/a | n/a | - Mean age at the time of primary ACL reconstruction was 21.6 6 3.5 years - 90% of skiers returned to their previous competition level, however, only 16-33% improved their world ranking by 3 years after ACL reconstruction compared to 60% in the control group - The pattern of secondary injuries alongside primary ACL ruptures showed little association with improved performance |
| **Müller et al. (2023)** | n/a | n/a | - The snow sport-specific rehabilitation challenges that need to be managed include a high degree of geographical decentralisation, seasonal climatic constraints, alternating off-snow and on-snow training, unique loading patterns with high forces and quasi-isometric-eccentric muscle actions, and regaining superior confidence to resume risk-taking in demanding situations - Sociocultural norms specific to snow sports entail a culture of sensation seeking, spectacle and heroism, further challenging rationally oriented pathways to return-to-sport - A potential solution to adequately address these issues could be applying a biopsychosocial and interdisciplinary approach to snow sports athletes’ RTS framework |
| **Müller et al. (2024)** | n/a | n/a | - Stakeholders reported needing more interprofessional cooperation and better information flow across the return-to-sport phases. - Expert networks, resources and structure strongly influence interprofessional cooperation and information exchange. - Athletes must be well informed and supported by a team of experts to have trust and confidence in the process. |
| **Nordahl et al. (2014)** | n/a | n/a | - Perceived facilitators to a successful return to competitive alpine skiing: self-belief, being mentally and physically prepared, regaining confidence in one's own ability, being given time and using active strategies - Perceived barriers to a successful return to competitive alpine skiing: negative feelings, for example, fear, disheartenment, a total lack of or ambivalent confidence in their own ability and the use of passive strategies |
| **Nwachukwu et al. (2017)** | n/a | n/a | - Patient-reported outcomes after ACL reconstruction among active athletes of different sports are comparable, whereas those of skiers are slightly poorer |
| **Praz et al. (2019)** | Immediate brace-free weight bearing with crutches, and active/passive knee range of motion exercises  Frequent quadriceps activation exercises  From 6 weeks to 3 months postoperatively, she participated in physical training under the supervision of a team of professional sports trainers | A milestone-based approach and qualitative progression monitoring | - Combined ACL repair with ALL reconstruction appears to offer important advantages for the patient with a quicker return to competition than ACL reconstruction - Rapid return to competition is likely multifactorial and related to the small size of tibial and femoral tunnels (4 mm), full preservation of the ACL stump and its mechanoreceptors, absence of donor site morbidity (no requirement for hamstring harvest) and the skier’s ability to start rehabilitation earlier - At 20 weeks postoperatively, the skier successfully participated in the slalom at the 2018 Olympic games |
| **Pujol et al. (2019)** | n/a | n/a | - The overall anterior cruciate ligament injury incidence was 8.5 per 100 skier-seasons; the primary anterior cruciate ligament injury rate was 5.7 per 100 skier-seasons - The prevalence of reinjury (same knee) was 19%. - The prevalence of a bilateral injury (injury of the other knee) was 30.5%. - Men and women were similar with regard to primary anterior cruciate ligament injury rate, career remaining after the injury - There were more anterior cruciate ligament injuries (primary, bilateral, reinjuries) among athletes ranking in the world top 30 |
| **Rosenstiel et al. (2019)** | n/a | n/a | - All athletes: combined ACL and ALL reconstruction is associated with excellent outcomes in professional athletes with respect to graft rupture rates, return to sport, knee stability, and reoperation rates after injury - Skiers: Contralateral ACL re injury in 10% of the skiers - Skiers: 88.9% returned to the same competition level as preinjury - Skiers: Average RTS after 7.8 months |
| **Smith et al. (2024)** | n/a | n/a | - Current RTS protocols primarily focus on biomechanical and neuromuscular factors in isolation, neglecting the important perceptual-motor-cognitive changes associated with ACL injuries and the high cognitive demands of skiing - Integrating perceptual-motor-cognitive considerations in testing and training progressions is key |
| **Spiess et al. (2019)** | n/a | Eccentric strength was measured during the Nordic hamstrings exercise performed on the NordBord hamstring testing system | - 49 ± 41 months post-surgery, relative total eccentric strength of both legs in the hamstrings of the ACL reconstructed group was significantly lower than in the healthy group - Leg asymmetry was significantly greater in athletes who had undergone surgery on one leg than in healthy subjects and athletes who had undergone surgery on both legs |
| **Stevenson et al. (1998)** | n/a | n/a | - 27% of the skiers reported a history of a knee injury - Female racers were 2.3 times more likely to have sustained a knee injury than male racers - 22% of the skiers reported an ACL injury - Females were 3.1 times more likely to sustain an ACL injury in comparison to their male counterparts - 22% of the ACL reconstructions failed, requiring additional surgery to the same ACL |
| **Strobel et al. (2001)** | n/a | n/a | - Such rare cause of a reflex extension loss due to femoral high noon graft placement has not been described previously and should be included as a differential diagnosis when evaluating patients with an extension deficit after ACL reconstruction |
| **Tecklenburg et al. (2007)** | RTS program focused on reducing pain and swelling, restoring ROM, and regaining strength | Clinical examinations performed at 3, 6, 9 weeks and 3 months post-operatively | - Patients with such a type of injury are most likely to have two surgical procedures: either meniscal repair and delayed ACL reconstruction or simultaneous meniscal repair and ACL reconstruction with the risk of a second arthroscopy due to scar formation |
| **Udry et al. (1997)** | n/a | n/a | - Information processing, reactive behaviour and coping attempts are key responses of skiers to season-ending injuries - Potential opportunities of season-ending injuries are personal growth, psychologically -based performance enhancement, and further development of physical-technical skills |
| **Verdonk et al. (2024)** |  |  | - It took three years before the patient was able to come back to competition - This case report highlights the complex challenges associated with knee surgery and rehabilitation in the context of sports injuries |
| **Westin et al. (2022)** | n/a | Side hop test; knee joint laxity measurement (KT-1000 arthrometer) | - A side-to-side difference in the side hop test and knee joint laxity may predispose an ACL reinjury in competitive adolescent alpine skiers |

ROM: range of motion; RTS: return-to-sport; RTP: return-to-performance; FIS: International ski and snowboard federation; ACL: anterior cruciate ligament; LEAP: lateral extra-articular procedure; EMG: electromyography; ALL: anterolateral ligament; KT-1000: specific type of arthrometer.
