# Supplementary material for: Returning to Performance After ACL Injury in Competitive Alpine Skiing: A Scoping Review and Evidence‐ and Expert‐Informed Practice Recommendations
Source: Scand J Med Sci Sports. 2026 Mar 8;36(3):e70246. doi: 10.1111/sms.70246 (PMC12968495; doi:10.1111/sms.70246)
Supplement: Supplementary file 2 — File S2: sms70246‐sup‐0002‐FileS2.docx. [file SMS-36-e70246-s003.docx]

**Supplemental File 2: Collection of Best Practice Ideas**

***Part 1 - "Neuromuscular Readiness"***

| **Exercise Category** | **Goals** | **Key Exercises** |
| --- | --- | --- |
| **Squat** | Enhance kinetic chain function and integration | **Ex. 1:** Safety-bar squat (**Early Rehab Phase**). *Focus: Back, front & side loaded; Progression: Bilateral to unilateral*  **Ex. 2**: RFE split squat with contralateral suitcase loading (**Early Rehab Phase**). *Focus: Back, front & side loaded; Progression: Bilateral to unilateral.* |
| **Leg Press** | Enable high-intensity loading of the lower limb extensor chain under reduced complexity | **Ex. 3:** 2:1 AEL leg press (**Late Rehab Phase**). *Focus: Mainly single leg loading using a 45° inclined leg press; Progression: Concentric-eccentric to eccentric-biased.* |
| **Hip Hinge** | Optimize hamstring loading across full muscle length through coordinated pelvic motion | **Ex. 4:** RDL in staggered stance (**Mid Rehab Phase**)*. Focus: Shoulder- & hand-loaded; Progression: Supported to partially supported.* |
| **Bridging** | Enable high-intensity loading of the hip extensors with established lumbo-pelvic control (A–P / Rot) | **Ex. 5:** SL hip thrust with heel-supported trolley (**Late Rehab Phase**). *Focus: Explore varied knee flexion angles to target glute–hamstring emphasis; Progression: Bilateral to unilateral & non-elevated to elevated shoulder.* |
| **Leg Curl** | Develop high-intensity eccentric strength and RTD of the hamstring muscles | **Ex. 6:** Manual leg curl with hip flexion bias (**Mid Rehab Phase**). *Focus: Eccentric-biased loading; Progression: Shortened to lengthened positions including tibial rotation.* |
| **Hip Abductors / External Rotators** | Enhance dynamic knee alignment through improved hip stabilization | **Ex. 7:** Banded lateral monster-walk with ISO Pallof press (**Early Rehab Phase**). *Focus: Conscious integration into multi-joint and complex movements; Progression: Isolated control to eccentric-biased loading.* |
| **Lower Leg** | Enhance ankle stiffness and distal control to optimize load transfer and stability | **Ex. 8:** Seated calf raise with Flywheel (**Mid Rehab Phase**). *Focus: Build up to plyometric and running mechanics; Progression: Isolated control to complex dynamic movements.* |
| **Deadbug** | Establish lumbo-pelvic control and proximal stability during dynamic limb motion (AE / AR) | **Ex. 9:** Deadbug bridging with alternating knee flexion (**Late Rehab Phase**). *Focus: Conscious integration into multi-joint and complex movements; Progression: Increasing lever length of arms and legs.* |
| **Weightlifting** | Improve explosive force production | **Ex. 10:** Reactive snatch balance or split jerk (**Late Rehab Phase**). *Focus: Mainly from hang position or blocks. Progression: Pulling to catching combined movements.* |
| **Isoinertials** | Enhance eccentric strength and deceleration control under variable resistance | **Ex. 11:** Flywheel deep squat catch (**Early Team Training Phase**). Focus: Optimize general force absorption control; Progression: Submaximal slow to submaximal fast to supramaximal fast. |
| **Ballistics** | Enhance reactive strength and neuromuscular control through stretch–shortening cycle loading | **Ex. 12:** Snap down squat jump onto box (**Mid Rehab Phase**). *Focus: Optimize rapid force absorption and recoil; Progression: Bilateral to unilateral & CON-only to reflexive eccentric and stretch-shortening cycle (slow to fast).* |

RFE: rear foot elevated; AEL: accentuated eccentric loading; RDL: Romanian deadlifts; A-P: anteroposterior; Rot: rotational; SL : single leg; RTD: rate of torque development; ISO: isometric; AE: anti-extension; AR: anti-rotation; CON: concentric; Ex.: exercise

***Part 2 - "Psychological Readiness and Social Support"***

| **RTP Phase** | **Goals** | **Key Exercises** |
| --- | --- | --- |
| **Post-Injury/Pre-Surgery**  Emotional stabilization and acceptance | Manage emotions and grief over loss of function; normalize reactions; conduct early mental-health screening (SMHAT-1); provide psychoeducation about recovery phases; build or activate social-support networks (family, peers, rehab staff). | **Ex. 1:** Emotional check-in routine – Daily self-reflection (3 emotions + 3 coping actions).  **Ex. 2:** Injury narrative reframing – Guided journaling on “what I can control/what I learned.” |
| **Post-Surgery/Early Rehab**  Confidence and engagement | Provide structured information to enhance perceived control; set short-term, achievable goals; integrate relaxation and breathing exercises for pain control; promote communication with coaches and teammates. | **Ex. 3:** Progress visualization – daily imagery of healing progress and next rehab step; additionally, use visualization to maintain and strengthen internal movement representations while physical activity is restricted.  **Ex. 4:** Mindfulness body scan – daily sensory-focus routine for pain regulation. |
| **Mid Rehab**  Motivation and autonomy | Facilitate autonomy in training tasks; implement progress tracking with visible milestones; train mindfulness and body awareness; encourage peer exchange with other recovering athletes. | **Ex. 5:** Set process goals – Focus on controllable daily actions (e.g., effort, attention, quality of movement) to sustain motivation and mastery.  **Ex. 6:** Autonomy-supportive task planning – Athlete co-designs or self-selects weekly rehab tasks to build ownership and intrinsic motivation. |
| **Late Rehab**  Coping and self-efficacy | Address fear of reinjury; use imagery and simulation of movement tasks; reinforce self-efficacy through positive feedback and mastery experiences; maintain team contact. | **Ex. 7:** Return-to-sport imagery – Guided mental rehearsal of sport-specific tasks and safe execution.  **Ex. 8:** Coping skills training – Practice coping techniques (breathing, self-talk, relaxation) to manage fear, frustration, and reinjury anxiety during late rehab. |
| **Early Team Training**  Confidence rebuilding and reintegration | Gradual exposure to training intensity; cognitive restructuring for risk perception; implement performance imagery and pre-performance routines; monitor motivation and stress. | **Ex. 9:** Challenge ladder – Athlete and coach co-create a hierarchy of sport-specific challenges of increasing complexity and risk to rebuild confidence and promote autonomy in progression. |
| **Regular Team Training**  Long-term robustness and self-reflection | Foster resilience and self-reflection; integrate mental toughness and focus exercises into training; encourage open dialogue on setbacks; maintain supportive coach–athlete communication. | **Ex. 10:** Resilience and reflection routine – Combine structured debriefs and reflective journaling to identify stressors, coping strategies, and growth experiences after challenging sessions, reinforcing long-term psychological robustness. |

RTP: return-to-performance; SMHAT-1: sport mental health assessment tool; Ex.: exercise.

***Part 3 - "Perceptual-Motor-Cognitive Readiness"***

| This document outlines ten perceptual-cognitive exercises distributed across three rehabilitation phases—Early Rehab, Mid Rehab and Late Rehab. These exercises progressively increase in motor demand, perceptual-cognitive load, and temporal pressure to simulate real-world performance conditions. | | |
| --- | --- | --- |
| **RTP Phase** | **Goal** | **Key Exercises** |
| **Early Rehab**  Controlled Environment | Restore movement quality and initiate perceptual-cognitive challenges in a low-load setting | **Ex. 1:** Reactive weight shifts with light stimuli - Athlete stands on a balance board or foam pad. Colored lights cue the athlete to shift weight in a specified direction; *Focus: simple reaction and attention switching; Motor demand: low; Temporal pressure: low; Progression: increase cue speed or shorten cue display time.*  **Ex. 2:** Step-and-reach with visual inhibition - A screen displays a sequence of colors; athlete steps forward only when a target color appears, inhibiting responses to distractors. *Focus: Inhibitory control and visual discrimination (emphasis on decision accuracy); Motor demand: low; Temporal pressure: low; Progression: increase cue speed or shorten cue display time.*  **Ex. 3**: Leg stability with dual task - Athlete maintains single-leg stance while answering simple cognitive tasks (math, recall). *Focus: Divided attention under postural demand to improve attentional control; Motor demand: low-moderate; Temporal pressure: low; Progression: increase cue speed or shorten cue display time.* |
| **Mid Rehab**  Dynamic Control | Reintegrate perceptual-cognitive processing into controlled dynamic movements and direction changes | **Ex. 4:** Reactive agility grid (low complexity) - Using a SpeedCourt or 3×3 grid, visual cues indicate movement direction. *Focus: Information processing speed; Motor demand: moderate; Temporal pressure: moderate; Progression: increase cue speed or shorten cue display time.*  **Ex. 5:** Inhibition step drill - Visual display randomly presents 'Go' or 'No-Go' cues. Athlete performs a 45° side-step cut only on 'Go'. *Focus: Response inhibition with emphasis on accuracy; Motor demand: moderate; Temporal pressure: moderate; Progression: increase cue speed or shorten cue display time.*  **Ex. 6:** Working-memory lateral shuffle - Lights display a 3-digit color sequence. Athlete memorizes and performs corresponding lateral movements. *Focus: Working memory and movement sequencing; Motor demand: moderate; Temporal pressure: moderate; Progression: Increase cue speed or increases sequence length.*  **Ex. 7:** VR-based change-of-direction training - Athlete reacts to virtual opponent movements or passing cues in VR. *Focus: Spatial anticipation and perception-action coupling under semi-realistic conditions. Motor demand: moderate-high; Temporal pressure: moderate; Progression: increase cue speed or shorten cue display time.* |
| **Late Rehab**  Ecological and Time-Pressured | Restore reactive agility and decision-making under sport-like temporal constraints | **Ex. 8:** Unanticipated 505 test with perceptual cue - Standard 505 change-of-direction test with direction cued late via light or visual display. *Focus: Fast decision-making under time pressure. Motor demand: high; Temporal pressure: high; Progression: increase cue speed or shorten cue display time.*  **Ex. 9:** Speed court multidirectional decision drill - Athlete reacts to randomized visual stimuli on the SpeedCourt, moving to illuminated targets. *Focus: Inhibitory control and working memory under temporal pressure (measures: reaction and accuracy); Motor demand: high; Temporal pressure: high; Progression: increase cue speed or shorten cue display time.*  **Ex. 10:** Sport-specific perception-action coupling (ball + cognitive load) - Athlete performs lateral side-steps while dribbling; direction changes based on unpredictable visual cues. *Focus: Real-time decision-making and dual-task motor control under high temporal pressure. Motor demand: high; Temporal pressure: very high; Progression: increase cue speed or shorten cue display time.* |

RTP: return-to-performance; Ex.: exercise; VR: virtual reality.

***Part 4 - "On-snow Progression"***

| **RTP Phase** | **Key Exercises** |
| --- | --- |
| **Mid Rehab**  Return-to-Snow –Restricted rehabilitation-team-led skiing with slalom skis | **Ex. 1**: Walking with skis on the flat  **Ex. 2**: Star steps with skis on the flat  **Ex. 3**: Scooter gliding on the flat  **Ex. 4**: Double pole gliding on the flat  **Ex. 5**: Skating on the flat  **Ex. 6**: Fall line skiing with alternating leg lifts or small jumps  **Ex. 7**: Back and forth (traverse skiing with alternating back and forth pivoting of the valley ski)  **Ex. 8**: Sideways sliding back and forth  **Ex. 9**: Sliding 360° turn after turning uphill  **Ex. 10**: Progression from plough turns, stem turns, and skidded parallel turns at low speed  **Ex. 11**: Shuffle (skidded parallel turns with alternating forward and backwards movements of the skis)  **Ex. 12**: Javelin (crossing of the inside ski over the outside ski while turning)  **Ex. 13:** While performing skidded parallel turns at low speed, consciously working breathing |
| **Late Rehab**  Return-to-Sport - Unrestricted coach-led skiing with slalom and giant slalom skis | **Ex. 14**: From skidded to carved turns with technical motion tasks for forth-back balance: skiing without poles, with open ski boots, and with one pole in front of and one pole behind the body  **Ex. 15**: From skidded to carved turns with technical motion tasks for lateral balance: e.g., holding the poles vertically or horizontally in front of the body, putting the outer hand at the hip – emphasizing body angulation, pressing the outer pole into the snow, pushing the inner knee with the hand turn inwards  **Ex. 16**: From skidded turns with technical motion tasks fostering unilateral loading: e.g., lifting the heel of the inside ski when initiating a turn, getting on and off the new outer ski when initiating a turn, single leg turns on the outer ski, single leg turns on the inner ski, single turns on one ski only  **Ex. 17:** Dynamic stop turns ("ice hockey stop")  **Ex. 18:** Carved turns: during the turn switch, pushing the skis alternately forward and backward  **Ex. 19:** Carved turns: slight jumps along the diagonal during turn switch  **Ex. 20**: Introducing dynamic skiing modes: e.g., 4 tuns fast–4 turns slow skiing, left–right turn variations, left foot RS ski–right foot SL ski, stop turns, turns with small jumps, dynamic short turns, trick turns  **Ex. 21**: Skiing with restricted visual, acoustic, sensory, and vestibular channels: e.g., skiing with tapped-up glasses, ear protection, short poles, leaving out left or right ski poles, low-cuff boots  **Ex. 22:** Flying turns (change of the direction dynamically from the old to the new outside ski)  **Ex. 23:** Consciously start the turn with weight on the toes, consciously finish it with weight on the heels |
| **Early Team Training**  Return-to-Competition – Competitive skiing with discipline-specific skis | **Ex. 24**: Dynamic trick turns with slalom skis  Obstacle parcour in the gates, slalom and giant slalom exercises using brushes  **Ex. 25**: Skiing in gates (all disciplines)  **Ex.** **26**: Competitive on-snow training under different conditions (snow conditions, visibility, terrain, course sets, equipment) and variations in timing, intensity, rhythm, and overall skiing volume.  **Ex. 27**: Unrhythmic and difficult courses and competition close setting with timing |
| **Regular Team Training**  Return-to-Performance – Full performance skiing | **Ex. 28**: Competition-like training: quality, intensity and risk taking  **Ex. 29:** One special run per training session as a focus to go “all in”  **Ex. 30**: Participation in level-relevant competition series: pushing the limits  **Ex. 31**: Event-specific training: top form at seasonal highlights |

RTP: return-to-performance; Ex.: exercise.
