# Supplementary material for: Returning to Performance After ACL Injury in Competitive Alpine Skiing: A Scoping Review and Evidence‐ and Expert‐Informed Practice Recommendations
Source: Scand J Med Sci Sports. 2026 Mar 8;36(3):e70246. doi: 10.1111/sms.70246 (PMC12968495; doi:10.1111/sms.70246)
Supplement: Supplementary file 3 — File S3: sms70246‐sup‐0003‐FileS3.docx. [file SMS-36-e70246-s002.docx]

**Supplemental File 3: Practical Experience Reports**

***by Christian Fink (orthopaedic surgeon)***

An anterior cruciate ligament (ACL) injury is, at best, a significant setback in the career of an alpine skier; at worst, it can be a career-threatening event. However, the positive aspect is that there have been numerous inspiring comebacks from such injuries, with some skiers achieving greater success than they did prior to their injury. This recovery process is a collaborative effort from the outset, and effective communication among all parties involved is crucial. The key participants included the injured athlete, the coach, the physiotherapist, the rehabilitation trainer, family members, and management, all of whom play vital roles in the athlete's well-being. In many instances, the surgeon is the first to deliver an unfortunate diagnosis to the athlete. Importantly, not all ACL injuries are identical. For a knee surgeon today, ACL injury itself often presents fewer challenges than associated injuries do. Frequently, injuries to cartilage, the meniscus, and additional ligaments determine the timeline and conditions for a skier's return to the snow. Therefore, a clear definition of ACL injury is of paramount importance. A straightforward classification system assisted me in aligning all participants in the rehabilitation group:

- - A: Isolated ACL injury
  - B: ACL injury with minor additional complications (e.g., small, uncomplicated meniscal tear, MCL sprain)
  - C: Complex ACL injury with accompanying ligamentous injuries, cartilage damage, or complex meniscal tears.

As the severity of the injury increases, so does the complexity of the surgical intervention, which can significantly influence the rehabilitation process. Prolonged periods of limited weight-bearing due to cartilage procedures or intricate meniscal repairs, as well as restricted range of motion resulting from additional ligamentous repairs, profoundly affect rehabilitation and recovery timelines. Upon assessing the extent of the injury, the surgeon can provide a general outlook on how the forthcoming months may unfold and, more crucially, how the upcoming days might proceed.

Athletes require a clear perspective early in their recovery. I emphasize to them that, in addition to coping with their current injury, they have the opportunity to increase their overall fitness and address long-standing limitations that have not prevented them from skiing but have hindered their performance (e.g., patellar tendinitis, lower back pain). I endeavour to share positive examples of skiers and other athletes who have successfully returned stronger than they were before.

I also prepare them for the reality that the rehabilitation process will not be a linear progression.

There will be fluctuations in progress, and such variability is normal rather than exceptional. Some athletes may have prior experiences with injuries; if these experiences are positive, they can be beneficial.

One of my patients, who had won several medals at the world championships just 1.5 years following an ACL injury, sustained a contralateral ACL injury three years later. Upon receiving this news, he looked at me and said, "Shit… but so what? We've done it once, so we can do it again. This time, it’s going to be an Olympic medal." This mindset is an excellent foundation for the rehabilitation process.

If an athlete has previously experienced negative experiences, we address potential modifications early on to induce greater comfort in the new situation (e.g., changing rehabilitation facilities or incorporating a psychologist). This initial phase is critical for laying a solid foundation for recovery, and the surgeon plays a significant role in this stage.

As the rehabilitation process progresses, the role of the surgeon ideally diminishes in importance.

However, I assure the athletes I treat that they "won't be rid of me" until they safely return to skiing and resume their competitive careers. This commitment means that I am available not only for regular follow-up visits but also for any unexpected issues that may arise (e.g., swelling or sudden onset of pain).

Surgeons should also participate in decision-making processes related to the escalation of training, scheduling the return to ski, and planning for early competitions. Throughout my medical career, and through the numerous years I have had the privilege of working with professional athletes, I have witnessed both remarkable comebacks and notable failures. Failures present mental challenges for everyone involved in a lengthy rehabilitation process, but they also provide valuable learning opportunities. Thus, it is essential to celebrate successes while learning from setbacks and remaining humble throughout the journey.

To me, TRUST is fundamental to a successful rehabilitation process. Trust is readily established when things progress positively but can be elusive in times of adversity. The athlete must trust the surgeon, and the surgeon must trust the physiotherapist; this trust must be reciprocal. The athlete should also have confidence in the individual (perhaps a coach or another athlete) who referred them to the surgeon. Additionally, they must trust the coach's and physiotherapist's recommendations regarding their return to sport, and so on. The most challenging situations I have encountered arose when lines of trust were compromised. Consequently, open communication and mutual respect for each other's expertise and commitment are highly important.

All participants involved in the rehabilitation process, including the media, must acknowledge that there are no miracles in medicine. Not every injury can be restored to a pre-injury state by surgical intervention, nor can any injection currently available promote cartilage regrowth or eliminate pain permanently. The "miracles" witnessed in professional sports are the culmination of hard work, risk-taking, and sometimes even the pain tolerance of the athlete. It is a privilege to work with professional athletes, but it can also be challenging in many respects. As a surgeon involved in the process of returning to sport (RTS), I can only pledge to the athlete that I will do my utmost, but I cannot guarantee the anticipated outcomes.

***by Marine Alhammoud (sports physician)***

Assessing skiers during the return-to-sport (RTS) process was my daily challenge as a Sports Medicine Physician (SMP) when I was working at the French Ski Federation. When an athlete wins medals after an RTS programme, this is a great success for the athlete as well as for the team doctor. However, I remember a promising European Cup female who sustained triple ipsilateral ACL injury. She was 18 years old and experienced recurring injuries during the 3-year follow-up period. The surgeries were all performed by the same expert lower limb surgeon. After the third surgery, despite a successful RTS programme, she quit her sports career because of a general shift in interest.

*Unidentified risk factors*

The first injury was attributed to a tactical mistake in Downhill (March 2015) due to a lack of experience in the choice of lines. The skier underwent bone‒patellar tendon‒bone surgery for a right ACL proximal rupture with a medial collateral ligament (MCL) grade 3 sprain. During the rehabilitation period, it was noted that she was eager to return to the “normal” ski training, willing to move on and perform serial ski runs, to compensate for the backlog and to be more competitive. This was quickly regulated by the staff several times; the coaches insisted on the necessity of completing the rehabilitation process. The second injury was due to a technical mistake during GS competition at the 9th postoperative month. A dipping terrain transition reduced the time the skier had to anticipate and adapt. She was out of balance and injured before she fell: a slip-catch mechanism was described. Graft revision was performed at the beginning of January 2016 with a four-strand hamstring tendon graft, lateral tenodesis, meniscal sutures and 2 anchors on the MCL tibial insertion. Six months after the second surgery, the RTS battery of tests was good, except for the ACL-RSI scale, which revealed psychological weakness. The rehabilitation team was oriented to improve self-confidence during skiing. The third injury seemed not understandable at first glance: the athlete fell during regular training on the first December 2016 and ruptured the ACL graft at the middle. The slope and snow conditions were good and easy, and the GS run was short (32 s). She had an enormous fall on a subtle terrain change. The coach did not understand what happened and mentioned that “she fell onto her skis and broke it, then all RTS tests would have been useless before…”. He also reported that she generally had “unidentified risk behaviour” and asked how to make her realize this “risky zone” to expose herself less. A second revision was performed in mid-January 2017 with a hamstring graft (semitendinosus of the left knee donor site), lateral meniscus suture, lateral tenodesis and MCL suture at tibial insertion.

*Looking back with the current knowledge*

During the third accident, the coach thought that the athlete experienced rapid fluctuations in distracted attention from skiing. The speed was not high, and no additional cognitive or motor stress was placed on the athlete. With the current knowledge, I assume that the combination of ACL reconstruction-associated neuroplastic alterations and skiing perceptual-cognitive demands contributed to the third injury, despite RTS testing suggesting that functional recovery has occurred. At that time, we did not have the tools to evaluate altered brain and neural activity that influence the perceptual‒motor cognitive (PMC) demands of skiing. Wondering what could I have done differently remains hypothetical because RTS protocols focused primarily on biomechanical and neuromuscular aspects that were not sufficient. When traditional landmarks were met, it was not acceptable to delay the RTS to work on “unidentified risky behaviours”. The 180° change in mentalities was initiated soon after this case by the Federation. The 9-month theoretical delay postoperatively was suppressed for the youth athlete, and no date was communicated until the “RTS passport” was validated with the mandatory steps. However, we did not integrate both motor and cognitive tasks during functional jump testing to determine dual-task cost in the clinic. In addition, the return-to-snow programme, which already includes drills challenging proprioception, should be further modified to increase the perceptual‒motor cognitive load. This will raise coaches’ and skiers’ awareness while on snow under various perceptual inputs and reactionary elements introduced one by one to start objectifying cognition and performance. Unfortunately, the present clinical case suggests that some athletes have paid the price of the gap in knowledge regarding how to best quantify PMC demands in alpine skiing.

*Most important lessons I learned*

The worst scenario is when no one understands nothing after an injury. In light of the current knowledge, the youth athlete is not “hot-headed” or “impulsive” but rather inexperienced with PMC elements. In contrast, a World Cup skier has naturally developed more skills related to proprioceptive tasks over time during skiing, which requires sensory processing under multiple cognitive stressors. I learned that the job of an SMP is not finished when functional recovery is achieved. The present case illustrates the need to integrate skiing perceptual-cognitive elements on top of the “green light” given by standard RTS testing. Age and time in the season are also crucial factors that ultimately influence decision-making and the timeline of clearance. I learned that it is difficult to protect young athletes from disturbing external influences. Talented young racers are also more vulnerable to the pressure of achieving the final goal of returning to competition as soon as possible. Financial restraints negatively impact young athletes who have no professional status (i.e., no sick leave, no salary).

*Recommendations for return-to-performance*

The RTS process needs to be individualised and flexible. After the surgical expectations are met, the rehabilitation framework, which is based on small steps, should focus on the following:

1. Sensory neuroplasticity and proprioception impairments:

To quantify motor performance and movement quality with or without cognitive load, a calculation for dual-task cost can be implemented. Equipment changes, helmets and goggles can be applied to hop testing, balance training, and closed chain strength exercises. Although it is difficult to ensure return to competition readiness, the on-snow progression should include drills (one element at a time) and evaluate responses before adding, for example, visual challenges or different ski lengths. It could help to give the skier the psychological and cognitive capacity to return to prior injury.

1. Apply a biopsychosocial and interdisciplinary approach:

The SMP should not hesitate to modulate the RTS timeline for youth athletes, who could benefit from additional measures to restore full self-confidence after ACL injury and psychological recovery instead of purely physical recovery. Interprofessional cooperation and information exchange could now be optimized to see “the big picture”, responding to the call for the application of a biopsychosocial and interdisciplinary approach to the RTS framework of snow sports athletes.

***by Alli Gokeler (physiotherapist)***

As a sports physiotherapist, I have long been driven by the challenge of safely and effectively returning athletes to their sport following anterior cruciate ligament (ACL) injuries. Early in my career, I focused heavily on measurable physical outcomes—strength and jump performance—as indicators of readiness to return to sport. This reductionist approach led to the development and publication of a standardized return-to-sport (RTS) test battery in 2016. However, through clinical experience and research, I came to realize that these tests, while reliable, failed to capture the complexity of real-world sport environments, where athletes must continuously perceive, decide, and act under pressure. ACL injuries, which often result from rapid cutting or pivoting movements, remain a major challenge in sports medicine. Despite surgical reconstruction aiming to restore stability, only approximately 55% of athletes regain their pre-injury performance level, and young athletes face re-injury rates of up to 40%. This discrepancy raised a critical question: Are we truly preparing athletes for the dynamic, cognitively demanding nature of sport?

Following my PhD, my postdoctoral research focused on addressing this gap by evolving RTS testing beyond isolated physical measures. The first phase refined existing RTS protocols, but their structured and predictable design limited their ecological validity. A turning point came with the adoption of an ecological dynamics framework, emphasizing the constant interaction among athletes, tasks, and the environment.

Subsequent studies have advanced this paradigm. We integrated inertial measurement units (IMUs) to capture athlete movement in realistic contexts and examined how cognitive load influences agility and coordination. The findings showed that decision-making demands significantly impair performance, highlighting the need to incorporate neurocognitive factors into rehabilitation. Subsequent work demonstrated that neurocognitive errors, such as lapses in attentional control or inhibitory functions, may contribute to ACL injury mechanisms themselves.

Building on these insights, my research team developed new RTS assessments combining physical and cognitive challenges—such as a motor stop-signal task and a neurocognitive hop test—to better reflect the multifactorial demands of sport. Comparative studies between computer-based and sport-specific tests further revealed that the ecological context profoundly influences how athletes respond to cognitive challenges. More recently, adding working memory components to change-of-direction tasks demonstrated that cognitive load directly affects performance and joint mechanics.

Collectively, this body of work represents a shift from traditional, standardized testing toward ecologically valid, cognitively integrated, and individualized assessments. Importantly, we must recognize that each athlete is unique—with distinct physical capacities, cognitive profiles, and contextual constraints. Therefore, rather than basing return-to-sport decisions on group averages, we must develop individual risk profiles that reflect each athlete’s personal strengths, vulnerabilities, and recovery trajectory.

My ongoing goal is to bridge the gap between laboratory testing and real-world performance, ensuring that athletes returning from ACL injury are not only physically ready but also cognitively, perceptually, and psychologically prepared to meet the true demands of their sport.

***by Jan Seiler (strength and conditioning expert)***

Personally, I’m a proponent of the early integration of general strength and conditioning during rehabilitation. The incorporation of neuromuscular stimuli, such as cross-education effects or overall strengthening of non-injured body parts, has always been an immediate priority in my approach to high-level athlete rehabilitation.

Nevertheless, I recognize that occasionally reducing the training load—despite not being fully supported scientifically—may be a sensible choice, particularly given the constant pressure athletes face to adhere to strict training and competition schedules. During the post-surgery phase, a well-planned cut-off can help maintain motivation for the upcoming, highly demanding phases, where increased training volume becomes possible. This decision is highly dependent on the individual circumstances at that particular moment in an athlete’s career.

This finding also underscores the importance of the athlete’s fitness state at the time of injury and rehabilitation. If an athlete is very well trained and has a robust physical foundation, missing a few weeks of structured training may have minimal impact and can even facilitate a quicker return to sport and performance. Furthermore, recognizing that returning to sport or performance within six months post-op is rarely realistic and often not the most sustainable approach, particularly with respect to reinjury risk, allows us to reduce the pressure of immediately compensating for lost training days at the very beginning of rehabilitation. Moreover, rehabilitation should be decoupled from fixed timelines and instead aligned with clear, evidence-based criteria.

Additionally, while nutrition plays a crucial role in supporting training adaptation, it is still too often neglected or not systematically integrated into the rehabilitation process. I am convinced that an evidence-based nutritional strategy can significantly support tissue healing, muscle rebuilding, and motor-cognitive function.

***by Matthew J Jordan (sports scientist)***

Early in my career as a strength & conditioning coach and sport scientist working with alpine ski racing, it became apparent that we lacked visibility on how alpine ski racers with ACL injury were tracking physically and psychologically after their return to snow and beyond as they pushed for a return to performance. This was problematic since ACL reinjury tended to occur ~ 18-24 months after the index surgery. After receiving medical clearance, the typical trajectory for a ski racer with an ACL reconstruction was to reach a basic level of physical fitness and then re-join the team for a short unstructured transition back to on-snow training. There were high expectations from both the team and the sport system that such an approach would result in a speedy return to the preinjury performance level. These athletes were not monitored after their return to snow in accordance with the severity of their neurophysiological knee injury. Instead, it was assumed that after passing return to snow criteria, the rehabilitation was complete and now the athlete should be encouraged and expected to make a return to performance.

However, achieving a return to performance on this unrealistic and aggressive timeline rarely occurred. The mismatch between expectations and reality, the lack of system-wide support and knowledge, and the absence of objective biomechanical/physical, psychological, social (biopsychosocial) monitoring contributed significantly to an unacceptably high prevalence of ACL reinjury that plagued many ski racers. There are profound consequences to a poorly managed ACL injury in ski racing. In some cases, this resulted in skiers with multiple (i.e., > 5) surgical procedures to address ACL re-injuries including to the contralateral limb injury. In other cases, ski racers reported catastrophic career ending subsequent ACL reinjury that resulted in debilitating post-traumatic knee joint osteoarthritis. After retirement, these individuals reported being robbed of their quality of life.

From a sport science perspective, a return to performance requires physical and psychological readiness to be tracked daily using objective assessments. We can adopt accepted practices from the science of athlete monitoring. This includes frequent (ideally daily) evaluation of training load and the training load reaction, athlete wellness, recovery and fatigue, and neuromuscular readiness. The sport scientist should be conscious of tracking the performance of both the affected and unaffected limbs after the return to snow transition to identify contralateral limb detraining. Additionally, it is crucial for the sport scientist to create the month-over-month, and year-over-year performance trajectory defined inside the boundaries of an expected performance funnel to identify a skier who is tracking faster/slower than or according to expectations. These analyses should be used in the context of an integrated support system (including the coach) to define realistic expectations and alleviate unnecessary psychological and physical stress. A conversation might include the statement: “Congratulations on achieving the milestone of returning to snow. Your hard work and dedication have served you well, but there is more work to do. All of us expect your physical, tactical, technical, and psychological function to continue to improve over the next several years. Give yourself the grace and the time to let your performance mature over the next few years. We will support you on this journey.”

This ongoing conversation must not occur in isolation. Instead, the sport scientist plays a vital role in leading an interdisciplinary approach that unifies the message in support of the athlete. The support team should be broad and include the medical personnel, the physical performance personnel, the nutritionist, the psychologist, and the coach. The sport-system (funding agencies, leadership) must consider how to fund and resource the management of ACL injuries in ski racing especially for the developmental level ski racer.

The sport scientist increases the value of this athlete-centred, integrated-support-system led approach by incorporating data into the daily conversation. Data can help account for confirmation bias, identify trends of progression and regression, and in can be used to better define the forecast for performance recovery and performance re-stabilization.

***by Philippe O Müller (sports psychologist)***

In my work as a sports psychologist, I have supported many athletes on their long and often uneven journey back to performance following an ACL injury. Over the years, one painful truth has become clear: psychological support often enters the process too late, usually only when problems have already emerged. Fear, frustration and loss of motivation are then viewed as symptoms that need to be "fixed" rather than as natural reactions that should be addressed proactively.

The early stages of rehabilitation, immediately following an injury or surgery, are usually dominated by medical and physical considerations. However, this is precisely when the psychological groundwork should begin. Emotional processing, grief over lost identity and the shock of disruption are rarely given adequate consideration. In my experience, athletes who receive early psychological support cope better throughout rehabilitation and experience fewer setbacks when the physical load increases.

Another recurring challenge is access. Many athletes and their parents are unaware of where to find sports psychology services or assume that they are needed only when something 'goes wrong'. In my view, sports associations and teams should ensure that athletes have structured access to mental health and sports psychology services as part of their medical and performance programmes. Psychological readiness should be treated as a standard component of return-to-performance programmes, not an optional 'luxury'.

On reflection, I realise that our field has sometimes placed too much emphasis on cognitive strategies such as self-talk, visualisation and reframing, while paying insufficient attention to emotional processing. Before athletes can effectively use mental tools, they often need to acknowledge and work through feelings of fear, anger or sadness. This emotional work lays the groundwork for genuine resilience.

With the knowledge I have today, I would address the issue of the fear of re-injury much earlier and in a more systematic way. In the past, this issue often arose only when athletes were about to return to the snow, at a stage when there was limited time and psychological space to work on it. I have learned that it is crucial to screen for fear and emotional readiness throughout the entire rehabilitation process, not just at the end. By identifying concerns early on, we can gradually implement suitable psychological interventions to help athletes regain trust in their body and confidence in their recovery before they take their first turn on the snow again.

In my experience, the most effective approach is a collaborative, autonomy-supportive approach. This involves treating athletes as active partners in shaping their rehabilitation goals, fostering realistic optimism and building a supportive network around them. Psychological readiness is an ongoing process: it develops continuously, phase by phase, similar to physical readiness.

***by Jörg Roten (on-snow coach)***

I am writing these lines based on my experience of nearly 20 years as a ski coach at the World Cup level.

*What are my most important lessons learned and recommendations regarding returning to performance?*

First and foremost, sufficient recovery time should be allowed after an anterior cruciate ligament injury in competitive alpine skiing. While every athlete and injury are unique, a common mistake is pushing too hard too early. Full knee function and physical fitness are essential before progressing to on-snow training and racing. The goal should be to train as much as possible—without compromising the knee’s recovery. Early on, it is crucial to balance therapeutic skiing, technical work, and gate training. This means that coordination and sensory function in the injured leg should be retrained while holistic and high-quality ski training should be maintained.

Second, clear and open communication between staff and a safe, supportive environment are key. Moreover, superior cooperation between the athlete, physician, physical therapist, off-snow coach, on-snow coach, ski technician, and other support team members is crucial, and one of these individuals should always take the lead. This role may change depending on the time frame. Initially, this will be the physician, followed by the physiotherapist and the off-snow coach; ultimately, when working on snow, this responsibility should be handed over to the on-snow coach. In any return-to-sport phase, the athlete should be put in the centre.

Third, in daily practice, it is often difficult for an on-snow coach to follow a precise plan. Working in the field is often dictated by slope conditions and weather. This is why I believe it is better to work towards goals than to necessarily follow a specific plan. Ultimately, the job of the on-snow coach is to implement and develop these goals in line with the conditions. The entire return-to-performance team must be flexible.

*Based on my experience, what works and what does not?*

1. Increasing intensity

With respect to the on-snow training intensity, the knee sets the pace. Accordingly, increasing intensity is not a linear process but should fluctuate below a predefined and medically and evidence-informed maximum load tolerance limit that increases over time. The intensity can be well regulated by skiing speed. It is clear that the load increases with skiing speed and that, particularly in early phases on snow, such speeds should be avoided. In contrast, at lower speeds, it is possible to focus on executing the movement correctly and protecting the joints. However, if the speed is too slow, care should be taken to avoid 'choking'.

1. Decreasing focus on the injured knee

At the beginning of on-snow training, the injured leg should receive special attention. In particular, the turn at which the injured knee is on the inside (extensive knee flexion in combination with loaded impacts) needs to be well controlled. Gradually, however, you should reach the point where both sides are treated equally. The focus on the injured leg should disappear over time so that both turns can be performed equally naturally.

1. Conscious choice of equipment

When one starts working on snow, one should begin with slalom skis. They are easier to handle. Subsequently, giant slalom skis can also be used. An improvement that I find beneficial is to vary these skis frequently and to ski with different skis on each leg. Edge tuning should be moderate, especially at the beginning of training, and under no circumstances should it be too aggressive or too sharp. The skis should be forgiving of small mistakes and should be prepared in such a way that they can be skied smoothly. Here, too, one can gradually approach a competition-like preparation. Good communication between the on-snow coach and the ski technician is necessary, whereas the on-snow coach should take the lead.

1. Improving sensory perception

What has proven very effective in practice is to deliberately reduce the perception of one or more visual, acoustic, sensory, and vestibular channels while skiing so that the athlete is forced to focus more on their feelings and on the movements themselves. At the beginning, this should be practiced only on well-groomed slopes that are not too busy or steep.

1. Varying/changing – practicing without doing the same thing twice!

As competitive skiers must be capable of performing a stable target technique under various constantly changing external conditions, varying and changing are essential; moreover, during on-snow training sessions, they are part of the various phases of the return-to-performance journey. This may include on-snow training under different conditions (snow conditions, visibility, terrain, course sets, equipment) and variations in timing, intensity, rhythm, and overall skiing volume.

*Looking back, what would I have seen/done differently with the knowledge I have today?*

First, experience often shows that, in the medium or long term, an athlete returns more strongly after fully recovering from their injury. Despite the tragedy of any ligament rupture, such an injury is always an opportunity and a chance to improve. This keeps focus, motivation, and confidence high at all times. In addition to working on the injured knee, there is an opportunity to work on technical details and seek perfection, especially in slow and sliding turns. I would place more emphasis on this aspect.

Second, close cooperation with all players, especially physiotherapists, is crucial. This is not always easy and requires understanding and effort from all sides.

Third, a comprehensive, detailed, and critical analysis of the accident with the aim of learning something from it is needed. There is always a reason for a crash or injury (critical conditions, loss of concentration, coaching errors, physical deficits, lack of fitness, imbalance, overpowering...).

***by Marlies Raich, formerly Schild (Athlete)***

My injury history:

- Bone avulsion of the anterior cruciate ligament (left)
- Meniscus rupture. The meniscus was sutured (left)
- Two arthroscopic meniscus operations were performed (left).
- Knee dislocation: posterior cruciate ligament, medial collateral ligament, capsule... anterior cruciate ligament intact. Since then, limited mobility in the left knee joint (left)
- Several arthroscopic procedures. Major problems: cartilage damage to the knee (left) and meniscus rupture
- Comminuted fracture of the lower leg and fracture of the tibial plateau (left)
- Medial collateral ligament rupture (right)

Immediately after my accidents, it was always important to be well cared for/supported. Fortunately, I had people around me whom I trusted 100% and who made the right decisions for me in situations where I was unable to make decisions myself. Receiving optimal surgical care was the first priority. During and after the surgery, it was important to treat the immediate symptoms, pain, and swelling and thus create an environment in which I could feel reasonably comfortable again, think clearly, and, above all, reduce or release my personal stress levels.

At the time of my accidents, I was often in top form and was looking forward to finally competing with others and showing what I had worked so long and hard for. I felt close to fulfilling my dreams and was full of confidence. Suddenly, it was all over. My goals were unattainable. There was a big question mark hanging over my future career. At the beginning, the most important step was to determine the facts. Regaining mobility and independence was always the first major goal. Creating a therapy plan to gradually return to a level of fitness that would allow me to lead a “normal” everyday life. In addition, finally, the actual goal: a comeback in skiing. It was a journey that was always very demanding, both physically and mentally.

A comeback consists of many ups and downs. Having structure in the form of a plan was important to me. Structure provides security, shows you the way forward, and enables you to set new goals. It is equally important to remain flexible when implementing the plan. Especially in rehabilitation, things do not always go according to plan, so I often had to find alternatives to what I had originally planned and strike out in new directions. I think you always have to be aware of that. The path to a comeback is not always straight. Of course, there are guidelines, but everyone has their own pace. To avoid major mental setbacks, it was important for me to keep an open mind and be flexible, seeing every change in direction as an opportunity for improvement. Often, you need to take a step back to move forward again.

Small steps toward a big goal: Having a goal is important and beneficial. In the case of a major injury, it is simply too far away and therefore seems unattainable at times. For me, it was important to focus on smaller goals that were achievable in each situation. It was essential to recognize the achievement of these goals, celebrate them internally as successes, and thus approach the big goal step by step. This took the pressure off and gave me more satisfaction in each phase. It also prevented me from taking too big a leap and overtaxing my body. Especially when I returned to team training, it was important to stick to my personal goals, increase them slowly, and unconsciously return to a competitive shape. It was important to be in constant communication with my coaches/trainers, to leave no questions or concerns unanswered, and to work together to set realistic expectations at each stage to avoid major disappointment.

Getting back on skis was always a major milestone, and it was important to simply take my time and regain my confidence step by step. Here, too, it was important for me to take it easy at first, not to compare myself to others, and to progress from easy to difficult at my own pace. I tried to choose the right moment for my return to team training. This meant easy conditions—and again, progressing slowly. Overdoing it made me feel bad, which in turn meant setbacks in my comeback plans. In the end, it is always a case of learning by doing. Trying, analysing, and constantly readjusting to finally be mentally and physically ready and full of confidence for competitions.
